# Generation and Evaluation of Modified *Opaque-2* Popcorn Suggests a Route to Quality Protein Popcorn

**DOI:** 10.3389/fpls.2018.01803

**Published:** 2018-12-06

**Authors:** Ying Ren, Abou Yobi, Leandra Marshall, Ruthie Angelovici, Oscar Rodriguez, David R. Holding

**Affiliations:** ^1^Department of Agronomy and Horticulture, University of Nebraska – Lincoln, Lincoln, NE, United States; ^2^Center for Plant Science Innovation – Beadle Center for Biotechnology, University of Nebraska, Lincoln, NE, United States; ^3^Division of Biological Sciences and Interdisciplinary Plant Group, University of Missouri, Columbia, MO, United States

**Keywords:** popcorn, QPM, *opaque-2*, lysine, maize-breeding, zein

## Abstract

Introducing traits from dent corn to popcorn is challenging because it is difficult to recover adequate popping characteristics. QPM (Quality Protein Maize) is a dent corn variety carrying the *opaque-2* (*o2*) mutation, specifying increased amounts of normally limiting essential amino acids, and modifier genes which restore the wild type vitreous kernel phenotype. In this study, we introgressed *o2* and selected for endosperm modification using vitreousness and high 27-kD gamma zein content. In this way, we recovered high-lysine, fully poppable Quality Protein Popcorn (QPP). BC_2_F_4_ individuals with vitreous kernels were confirmed to be *o2* mutants by both genotyping and SDS-PAGE. Amino acid profiling of BC_2_F_4_ individuals showed that they all have significantly increased lysine compared with popcorn parental lines. Principal Component Analysis of the amino acid profiles showed that all introgressions were grouped with corresponding QPM parental lines. Popping analysis of the BC_2_F_5_ individuals showed that while there is variability in popping volume between lines, some lines show equivalent popping to the popcorn parent. In this proof-of-concept study for QPP, we have shown that it is possible to rapidly recover sufficient popcorn characteristics in a modified *o2* background using simple phenotypic, biochemical and genetic selection. Furthermore, this shows increased γ-zein is an acceptable substitute for α-zein for full poppability. Since we have developed multiple QPP introgressions, this gives good scope for ongoing hybrid production and future evaluation of agronomic performance and selection of elite hybrids. In a wider context, this study shows the potential for breeding beneficial traits into popcorn for agronomic improvement.

## Introduction

Maize a major cereal crop ranking number one is in the world in grain production. Like other cereals, it is nutritionally imbalanced, as the dominant seed storage proteins, the zeins, are devoid of two essential amino acids, lysine, and tryptophan. Compared with the optimal amount required for human nutrition which is 5% lysine and 1.1% tryptophan, respectively, maize grain contains only 1.5–2.5% lysine and 0.25–0.5% of tryptophan (Young et al., 1998). The *opaque-2* (*o2*) mutant which has kernels with reduced zein content and increased lysine content (Mertz et al., 1964) offered potential as a more complete source of plant protein. However, *o2* mutant kernels have soft endosperm, increased susceptibility to fungal pathogens and mechanical damage and grain yield is reduced. Quality Protein Maize (QPM) was developed by breeding *o2* mutants with normal kernel hardness and vitreousness while retaining the high lysine content. The nutritional benefits of QPM were confirmed in feeding trials (Sofi et al., 2009; Panda et al., 2010; Mbuya et al., 2011).

Several QPM conversions have been performed in dent corn (Babu et al., 2005; Manna et al., 2005; Collard and Mackill, 2008; Surender et al., 2017). Introgression of the *opaque-2* gene without modifier genes into popcorn backgrounds was carried out in several studies where popping was not maintained (Zhou et al., 2016; Adunola, 2017). To date, there have not been any reports of QPM conversion for popcorn lines that have maintained popping. In fact, introgression of any dent corn trait to popcorn was rarely reported. Dent corn × popcorn crosses were made mainly for mapping studies of quality trait QTLs for popcorn improvement such as higher yielding hybrids and popping expansion volume (PEV) (Dofing et al., 1991). The development of Quality Protein Popcorn (QPP) is challenging because it requires full QPM conversion but with the added challenges resulting from popcorn maintaining *Ga1-s* locus and recovering acceptable popcorn quality.

Breeding for QPM requires introgression of both the *o2* allele and multiple unlinked modifier quantitative trait loci (QTLs). Availability of *O2* in-gene and flanking markers can facilitate the introgression process of the *o2* allele (Babu et al., 2005; Babu and Prasanna, 2014; Surender et al., 2014; Kostadinovic et al., 2016; Krishna et al., 2017). The difficulties encountered in QPM conversion projects result from restoring kernel vitreousness because of the complexity of modifier QTLs. Several mapping studies were carried out and QTLs for modifiers (in terms of kernel vitreousness and density) were mapped on Chromosomes 1, 5, 7, and 9 (Holding et al., 2008, 2011; Babu et al., 2015). Multiple lines of evidence suggest that an increase in gene expression and protein accumulation of 27-kD γ-zein plays a major role in modification in QPM and this gene resides within the major QTL on Chromosome 7 (Geetha et al., 1991; Holding et al., 2008; Wu et al., 2010; Holding, 2014). The characteristic increase has been used as a biochemical marker for endosperm modification in QPM. Recently, it was verified that the 27-kD γ-zein protein increase is conferred by a duplication event at 27-kD γ-zein locus (Liu et al., 2016). Causal genes within other QTLs remain unknown. As a result, QPM conversion still relies heavily on phenotypic selection of endosperm modification although the 27-kD γ-zein genetic and biochemical markers are very useful.

Comparison between the B73 genome and the popcorn landrace Palomero Toluqueno revealed that Palomero genome is ∼ 22% smaller yet with larger predicted gene number being around 58,000, compared with 50,000 for B73 (Walbot, 2008; Schnable et al., 2009; Vielle-Calzada et al., 2009). Popcorn usually carries cross-incompatibility genes (known as gametophyte factors) that prevent popcorn from being fertilized by pollen of dent corns. This has been exploited for propagation of popcorn since it need not be grown in isolation from transgenic hybrid corn. Maintenance of *Ga1-s* is required in popcorn breeding programs. Additionally, breeding for popcorn improvement and trait introgression into popcorn germplasm requires maintenance of PEV. Studies mapping PEV in several dent × popcorn populations indicate multiple and variable QTLs (Dhliwayo, 2008; Li et al., 2007, 2009) and show the complexity of PEV. Introgression of dent traits into popcorn backgrounds is hindered by dent alleles with negative effects on PEV.

A previous QPM breeding program adopted both foreground (for *o2*) and background selection using MAS (marker-assisted selection) and developed QPM conversion lines within two backcrossing generations (Babu et al., 2005; Kostadinovic et al., 2016). MAS has not been widely used in selection of modifier genes because regions delineated as modifier QTLs tend to be large genetic intervals and highly variable in effect. The most significant QTL, 27-kD γ-zein duplication, is common across all QPMs and can now be used as a foreground marker (Liu et al., 2016). Also, the fact that mapping studies of modifier QTLs had been limited to specific QPM germplasms, might suggest that other minor effect QTLs are not common across all QPMs. This may restrict the application of MAS in projects such as this one in which multiple introgressions from different QPMs were performed.

In this study, we initiated QPP development with three QPMs and 11 popcorn lines. We aimed to introgress *o2* from QPM to popcorn germplasms while maintaining *Ga1-s*, endosperm modification, popcorn kernel shape and popping ability. Since the correlation between kernel vitreousness and popping expansion is high (Hoseney et al., 1983; Erazo-Barradas, 2009), selection on the basis of kernel vitreousness was expected to result in simultaneous selection for PEV. Analysis of resultant BC_2_F_4_ and BC_2_F_5_ populations including lightbox selection, zein profiling, amino acid profiling, and quantitative popping tests were carried out. We developed multiple QPP inbreds from which hybrid production is now in progress. These results represent a breakthrough in the introgression of traits from dent corn into popcorn and show the general potential for popcorn improvement.

## Materials and Methods

### Plant Materials

The 11 elite popcorn lines, whose identities are withheld, were provided by ConAgra. For QPM lines, K0326Y, is a tropical QPM inbred line developed in South Africa by Hans Gevers (Gevers and Lake, 1992). The other 11 QPMs were from North Central Regional Plant Introduction Station.

### Total Zein Extraction

Zeins and non-zeins were extracted according to an established method (Wallace et al., 1990). Briefly, kernels for analysis were ground into flour. 50 mg flour was incubated overnight in 1 ml borate extraction buffer (with 2% β-mercaptoethanol) with shaking at room temperature. After centrifugation at room temperature for 15 min, 300 μl supernatant (total protein extract) was used for precipitation of non-zeins by addition of ethanol to final concentration being 70%. Resultant supernatant (zein protein solution) after centrifugation was dried in a SpeedVac and resuspended in 200 μl of ddH_2_O. 5 μl was used for SDS-PAGE analysis of zein profile.

### DNA Isolation

Leaf tissue was collected from individuals in backcrossing generations (BC_1_ and BC_2_) for DNA extraction. DNA extraction was carried out using a BioSprint 96 workstation from Qiagen according to the user manual.

### Genotyping Using *o2* in-gene Marker umc1066

Polymerase Chain Reaction (PCR) was carried out in 20 μl volume consisting of NEB Taq 0.15 μl, 0.2 μM dNTPs, 2 μl standard Taq Reaction Buffer (10×), 0.2 μM forward/reverse primer of umc1066, template DNA 50 ng. PCR procedure was as follows: initial denaturation at 94°C for 4 min followed by 35 cycles of three steps including 94°C for 30 s, 57°C for 60 s, 72°C for 60 s. Final elongation was done at 72°Cfor 10 min. PCR products were visualized on 3∼4% agarose gels. Individuals with *O2o2* genotype were selected in backcrossing generations. This was also used for confirmation of *o2* mutants in later generations (BC_2_F_2_, BC_2_F_3_, and BC_2_F_4_).

### Evaluation and Selection of Endosperm Modification

Kernels were put embryo side down on a light box. Based on arbitrary visual estimation of the opaque endosperm proportion, kernels were classified into five types with Type I being fully vitreous, Type II being ∼ 25% opaque, Type III being ∼ 50% opaque, Type IV being ∼ 75% opaque and Type V being fully opaque.

### Amino Acid Profiling

Samples for amino acids profiling analysis were ground together into a fine flour. Free amino acids (FAA) were extracted from 6 to 7 mg of the flour pooled from 3 kernels of 3 QPM lines, 4 popcorn lines and the six BC_2_F_4_ introgressions, while protein-bound amino acids (PBAA) were extracted from 3 to 4 mg. FAA were extracted and analyzed as described (Angelovici et al., 2013) using a UPLC-MSMS system (Xevo TQ-S from Waters Corporation). For the analysis of protein-bound amino acids (PBAA), acid hydrolysis (Fountoulakis and Lahm, 1998) was performed prior to the FAA extraction and LC-MS/MS analysis described above. Briefly, 200 μl of 6N HCl was added to ∼4 mg of flour and incubated for 24 h at 110°C. Ten micro liters (10 μl) were taken from the hydrolyzed samples and dried using a Savant SpeedVac concentrator (Fisher Scientific) before resuspension in the FAA extraction buffer and further analyzed as described (Angelovici et al., 2013).

### Quantitative Measurements of Pop Volume

BC_2_F_5_ samples along with corresponding popcorn parents were prepared and adjusted to same moisture content for quantitative popping. After popping treatment in an Orville Redenbacher Hot Air Popcorn Popper, a 200 ml cylinder was used to measure the total volume.

### Total Protein Extraction and Relative Quantitation

Total protein extract (as described in Total Zein Extraction) from 50 mg kernel flour were diluted 250 times and 25 μl of the diluted solution were used for Bicinchoninic Acid (BCA) protein assay. Protein concentration was assessed by the BCA assay kit (Pierce) according to the user’s manual. Absorbances at (OD = 562 nm) were measured on a microplate reader Synergy2 (BioTek). Protein concentrations were determined from the BSA standard curve. Protein concentrations were compared between BC_2_F_4_ QPP introgressions with corresponding popcorn parents.

### Statistical Analysis

Data were presented as Means ± SEM. *P*-values less than 0.05 were considered significant. Principal Component Analysis (PCA) was carried out using R software.

## Results

### Quality Protein Popcorn Breeding Scheme

**FIGURE 1:**
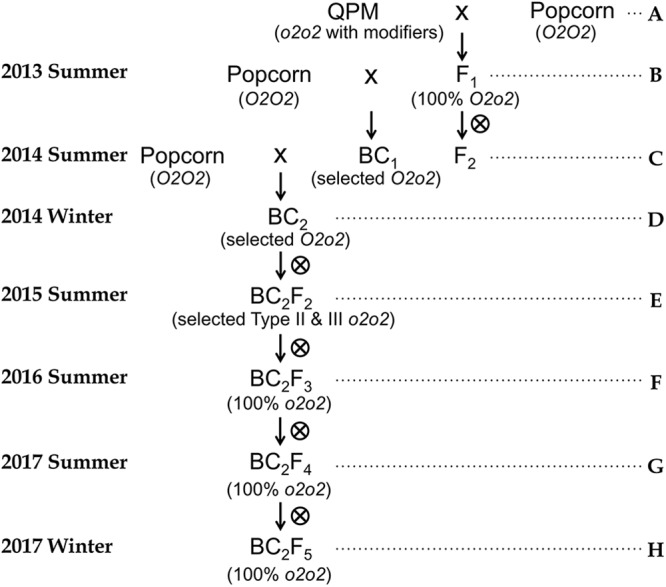
Breeding scheme for quality protein popcorn. A through H represent the steps used. **(A)** Selection of *Opaque-2* polymorphic markers between QPM and popcorn parental lines; Make F_1_ crosses uni-directionally using popcorn as male parents. **(B)** Confirm that umc1066 is a co-dominant type of marker that can be used to differentiate *O2O2*, *O2o2*, and *o2o2* genotypes; Simultaneously self F_1_ and backcross it to popcorn parental line (recurrent parent) to get F_2_ population and BC_1_ population. **(C)** Assess modifier transfer using F_2_ which segregates 25% *o2*. Select vitreous popcorn-like kernels to identify *o2* mutants by SDS-PAGE and determine whether they can pop at F_2_ stage. **(D)** Identify BC_1_ individuals with *O2o2* genotype in the BC_1_ generation; Carry out another generation advancement by backcrossing *O2o2* BC_1_ to popcorn parental lines to get BC_2_ population. **(E)** Identify BC_2_ individuals with *O2o2* genotype and advance them to BC_2_F_2_ population; Select Type II and Type III opaque kernels which are expected to be *o2* mutants. Randomly select several such kernels to do both genotyping and SDS-PAGE zein profiling to confirm they are *o2* mutants. Self-pollinate selected Type II and Type III opaque kernels to BC_2_F_3_ generation. **(F)** Select BC_2_F_3_ individuals with complete modification. Carry out genotyping and SDS-PAGE on completely vitreous kernels to verify they are modified *o2* mutants. For verified *o2* mutants with complete modification, advance by self-pollination to BC_2_F_4_ generation. **(G)** Ears were selected by the following two criteria: 1. Ear characteristics similar to popcorn parents. 2. Uniform modification of kernels across the whole ear. Perform amino acid profiling and preliminary popping analysis on kernels from selected ears. **(H)** Bulk up seeds for formal popping analysis.

Three QPM germplasms were selected as *o2* donors which are CML 154Q, Tx807, and K0326Y (Gevers and Lake, 1992) (Supplementary Figures S1, S2 and Supplementary Table S1). Zein profiles of three QPMs and 11 popcorn lines (labeled as P1–P11 to preserve identity) are shown (Supplementary Figure S3). We initiated the introgression with two continuous backcrossing generations followed by four selfing generations (Figure 1). Because of the presence of *Ga1-s* in popcorns and *ga1* in most dent corns, popcorns had to be used as male parents for cross initiation. Genotyping was carried out for *o2* polymorphism across popcorn, QPMs and F_1_ crosses (Supplementary Table S2 and Supplementary Figures S4A,B). For the purpose of maintaining *Ga1-s*, the resultant F_1_s were backcrossed onto respective popcorn lines (recurrent parents). F_2_ populations were generated for evaluation of modifier transfer across different crosses (Supplementary Table S3). A scale for endosperm opaqueness was devised using a light box as described (Vivek et al., 2008; Supplementary Figure S5). From the *p*-value, the ratio of opaque to vitreous kernels in these F_2_ ears deviates significantly from 1 to 3 implying that some *o2* mutant kernels are fully or partially modified. F_2_ populations with small *p*-value were promising for modifier transfer. Small roundish popcorn kernels were observed in F_2_ populations. Popcorn phenotype vitreous kernels were selected for both SDS-PAGE analysis and preliminary popping analysis to verify the presence of fully modified popcorn-like *o2* mutants in the F_2_ population (Supplementary Figure S6). A proportion of F_2_ popcorn-like kernels that were fully vitreous were obviously modified *o2* from their zein profiles. Furthermore, when such popcorn vitreous kernels were heated, 100% (including 25% *o2/o2*) popped showing proof-of-concept with only a single dose of popcorn. Individuals in backcrossing generations (BC_1_, BC_2_) carrying the *o2* allele were screened by genotyping using umc1066 (Supplementary Figure S4C). BC_2_F_2_ kernels harvested were assigned a level of modification on a scale defined in the F_2_ population (Supplementary Figure S5). In terms of genotype, they can be *o2* mutant with complete modification which is ideal. However they can also be heterozygous (*O2o2*) or homozygous (*O2O2*) with wild type phenotype. In order to guarantee the presence of *o2o2* genotype for later generation advancement without the need to genotype all kernels, we selected Type II and Type III semi-opaque kernels from BC_2_F_2_ populations for SDS-PAGE zein profiling (Supplementary Figure S7). After Type II and Type III kernels were confirmed to be *o2/o2* mutants, they were advanced into BC_2_F_3_ and BC_3_ populations. In the BC_2_F_3_ generation, ears with good modification were selected and used for characterization of completely vitreous popcorn-like *o2* mutants (Supplementary Figure S8). Candidate lines were advanced to the BC_2_F_4_ generation. Analyses of the resultant BC_2_F_4_ and BC_2_F_5_ were carried out and provided proof of concept for the conclusion that QPP is achievable using the breeding strategy reported here.

### Confirmation of *o2* Genotype in BC_2_F_4_ Population With Full Modification

**FIGURE 2:**
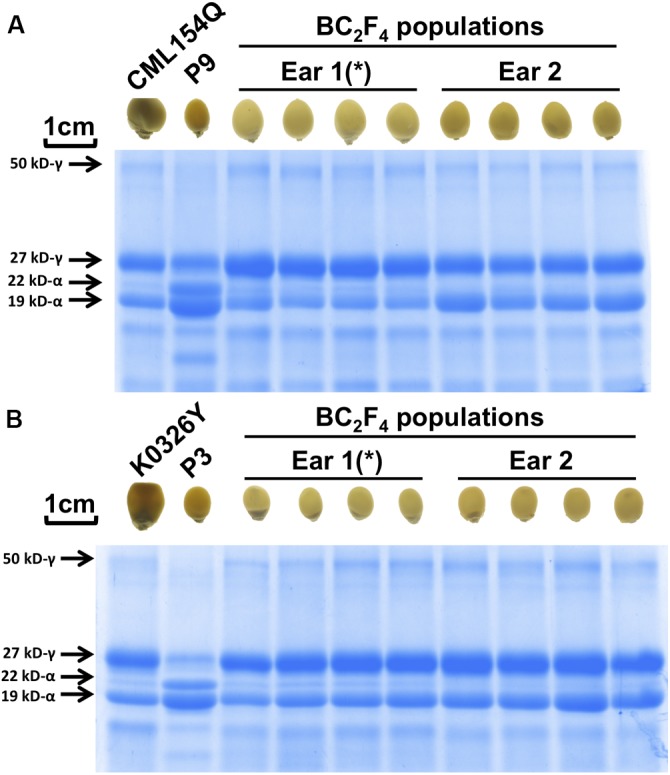
SDS-PAGE confirmation of modified *o2* mutants in uniformly vitreous BC_2_F_4_ population. **(A)** CML154Q introgression using P9. **(B)** K0326Y introgression using P3. The introgression marked with asterisk (^∗^) was selected for later amino acid profiling analysis. Confirmation of other *o2* introgressions were shown in Supplementary Figure S9.

**FIGURE 3:**
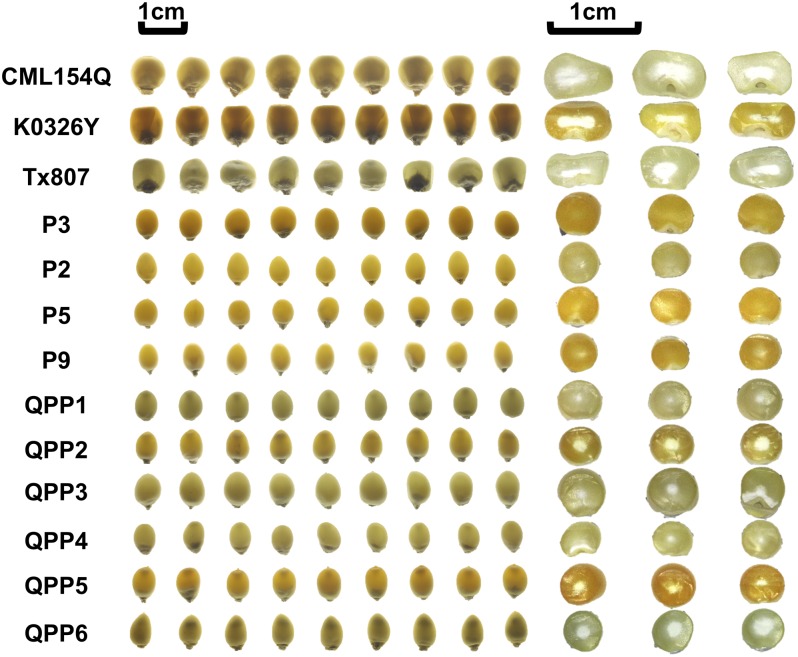
QPM, popcorn and six BC_2_F_4_ introgressions prepared for amino acid profiling; Left, kernel modification on Light box; Right, kernel dissection; QPP1, introgression from cross K0326Y × P3; QPP2, introgression from cross CML154Q × P2; QPP3, introgression from cross CML154Q × P9; QPP4, introgression from cross K0326Y × P3; QPP5, introgression from cross K0326Y × P5; QPP6, introgression from cross Tx807 × P2.

We selected promising BC_2_F_4_ ears with uniform modification (Figure 2 and Supplementary Figure S9A). SDS-PAGE gel analyses (Figure 2 and Supplementary Figure S9B) and umc1066 genotyping results of BC_2_F_3_ (Supplementary Figure S8B) confirmed that all kernels were modified *o2* mutants. Consistent with their vitreous appearance, they all have increased 27-kD γ-zein protein with respect to the wild type (popcorn parent) control. The uniformity in enhanced expression of 27-kD γ-zein implies that phenotypic selection alone is sufficient for the retention of this modifier QTL. BC_2_F_4_ ears from selected modified Type I *o2* kernels show more uniform modification than the BC_2_F_3_ population, where there was more variability for modification in the ears. Because of lack of kernels for the Tx807 introgression which is also at an intermediate stage of modification restoration (Figure 3), it is not included for SDS-PAGE confirmation here (but included in later amino acids profiling to see the lysine difference).

### Amino Acids Profiling Revealed High Lysine in All Six BC_2_F_4_ *o2* Introgressions

In order to determine whether these popcorn-like *o2* mutants have the high quality protein phenotype, amino acid profiling (both protein-bound amino acid and free amino acid) was carried out for six introgressions and parental lines including three QPM parents and four popcorn parents (Fountoulakis and Lahm, 1998; Angelovici et al., 2013). We also included B73 wild type and B73*o2* as dent corn controls for comparison to modified BC_2_F_4_ samples. Vitreous endosperm content for the profiled kernels was shown by both light transmittance and by cutting kernels transversely (Figure 3). Here we included Tx807 BC_2_F_4_ individuals, whose endosperm modification was not as advanced as other introgressions. Compared with wild type popcorn parental lines (P3, P2, P5, P9), all *o2* mutants (QPMs, QPP introgressions) have a small opaque center. Compared with QPP1-QPP5, the amount of the central opaque region of QPP6 is larger. This is consistent with the fact that it is only partially modified currently and requires selection in future generation advancements. QPP1 and QPP4 had the least central opaque region, consistent with their vitreous appearance. For QPP3 and QPP4, the kernels had a slightly altered distribution of the opaque region. In total, sixteen amino acids were detectable and quantified for each sample. The nine kernels shown for each genotype represent three pools of three kernels (three biological replicates). The sixteen amino acids measurable were Ala, Arg, Aspx, Glx, Gly, His, Ile, Leu, Lys, Met, Phe, Pro, Ser, Thr, Tyr, and Val (Supplementary Table S4) where Glx is both glutamic acid and glutamine.

**FIGURE 4:**
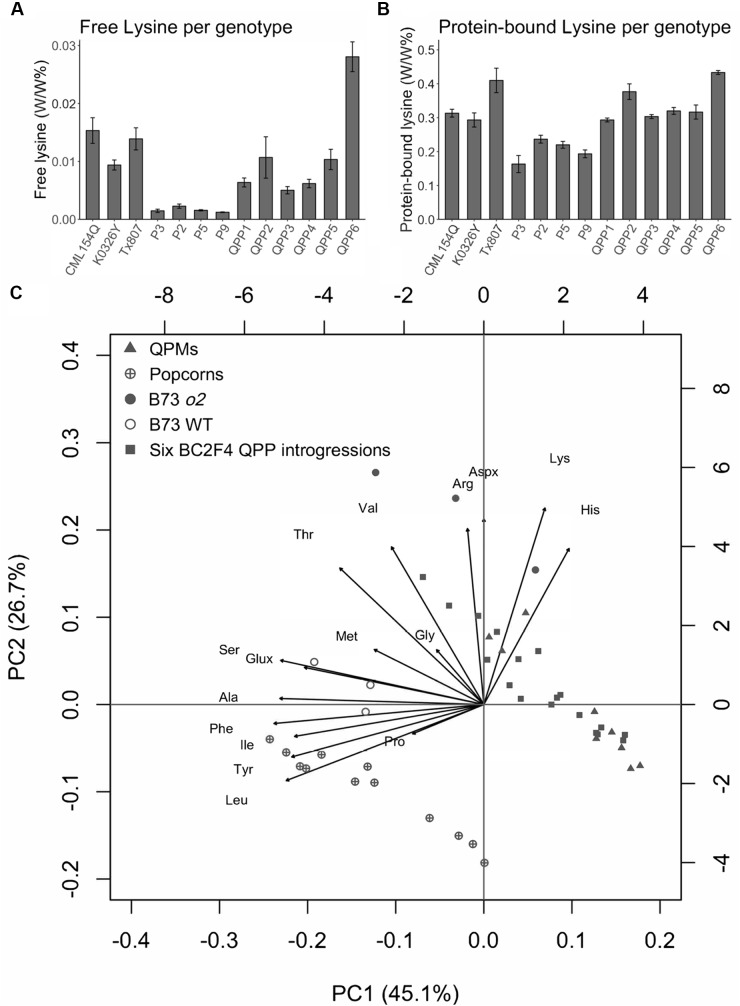
Confirmation of high protein quality of six QPP BC_2_F_4_ introgressions in comparison to QPM (CML154Q, K0326Y, and Tx807) and popcorn (P3, P2, P5, and P9) controls. **(A)** free lysine comparison. **(B)** protein-bound lysine comparison. **(C)** PCA analysis of protein bound amino acids. Principal component scores (PC1 and PC2) from each observation were plotted as dots in different shapes. Amino acids (variables) were plotted as arrows.

The results showed that all six introgressions have significantly increased content of both free lysine and protein-bound lysine compared with corresponding popcorn parents (Figure 4). The ratio of protein-bound lysine between QPPs and corresponding popcorn parental lines ranges from 1.45 fold (QPP5 compared with P5) to two-fold (QPP4 compared with P3). For free lysine, the ratio ranges from 4.05 fold (QPP3 compared with P9) to 12.3 fold (QPP6 compared with P2) (Supplementary Table S4). To extrapolate and display the global variation for all amino acids, we adopted the Principal Component Analysis (PCA) used in multivariate analysis (Figure 4C and Supplementary Figure S10). PCA using protein-bound amino acids data showed that the six introgressions grouped together with QPM germplasms in a distant group from popcorn parental lines (Figure 4C). This result suggests that the six introgressions have amino acid profiles similar to QPM. From the PCA biplot, the 15 amino acid (15 variables indicated as vectors) have different coordinations, implying different contribution to the total variation. Among the ones pointing toward the *o2* group, four amino acids (Lys, His, Aspx, Arg) were positively correlated with *o2* lines, whereas Leu, Tyr, Ile, and Ala were positively correlated with wild type germplasms. PCA analysis of free amino acids was also carried out and no clear pattern was observed across different *o2* mutants (QPM parents, QPPs, and B73*o2*) (Supplementary Figure S10). Previously, whole kernel amino acids contents were compared between *o2* and wild type maize and showed the general lysine increase resulting from globally increased non-zein proteins (Hunter et al., 2002). Here, we compared the ratio of *o2* by wild type for 15 amino acids measured both in the above study and this study to see whether the above eight amino acids showed similar pattern between all *o2* and wild type samples (Supplementary Figure S11). This showed that the log2 value of the ratio of amino acid (*o2* by wild type) is consistently positive for Lys, His, and Arg, suggesting there is more of these amino acids in *o2* background than in wild type backgrounds. In contrast, the log2 value of the ratio of amino acid (*o2* by wild type) was consistently negative for Met, Ser, Glx, Ala, Ile, Leu, and Phe. In both PCA biplot and comparative analysis of protein bound amino acid profile, Leu, Ile, Ala stand out as amino acids that are reduced in the *o2* background compared with wild type. Results of BCA protein assay on BC_2_F_4_ populations indicated that there was no reduction in total protein in all six QPP germplasms (Supplementary Figure S12). Specifically, for QPP3 and QPP4, significant increases of total protein (α = 0.05) was observed compared with corresponding popcorn parental lines.

### Selection of Fully Modified *o2* Popcorn Can Result in Equivalent Popping to Popcorn Parents

**FIGURE 5:**
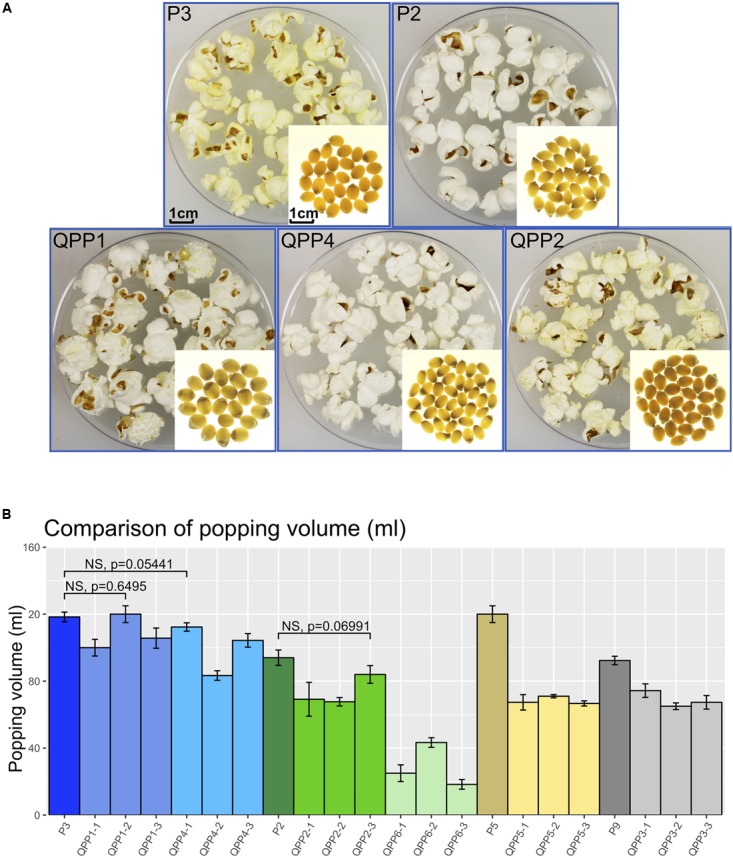
Comparison of popping characteristics between QPP introgressions with corresponding popcorn parents. **(A)** kernels before and after popping (QPP1, BC_2_F_4_ introgression from cross CML154Q × P3; QPP4, BC_2_F_4_ introgression from cross K0326Y × P3; QPP2, BC_2_F_4_ introgression from cross CML154Q × P2. **(B)** Popping volume comparison between QPPs and corresponding popcorn parental lines. Brackets between a popcorn parent and a QPP progeny indicate no significant differences.

Small scale qualitative popping analysis on BC_2_F_4_ kernels confirmed them to be fully poppable (not shown). After further bulking, BC_2_F_5_ kernels were used for small scale quantitative popping analysis (Figure 5A). To represent a general popping ability, three ears were selected which were the progeny of the BC_2_F_4_ ears used for amino acid profiling. Three pools of kernels (3.33g each) were used as biological replicates for each ear. Percentages of popped kernels were above 90% for all QPP and Popcorn parental lines (Supplementary Table S5). Volume of the popped kernels were measured and used for pair-wise comparison with corresponding popcorn parental line (Supplementary Table S5 and Figure 5B). Comparisons that exhibited no significant difference are highlighted in bold in Supplementary Table S5 and bracketed in Figure 5B. There were no significant differences in the popping volume scores for QPP1-2 vs. P3 comparison [*t*(3,2) = 0.50, *p* = 0.6495], QPP4-1 vs P3 comparison [*t*(3,93) = -2.71, *p* = 0.0544] and QPP2-3 vs. P2 comparison [*t*(3,92) = -2.47, *p* = 0.0699]. Popping volumes equivalent to corresponding popcorn parents were achieved in these BC_2_F_5_ populations (QPP1-2, QPP4-1, QPP2-3). For QPP1 and QPP4, where comparable popping volume was observed, the least residual proportion of opaque endosperm was observed. For QPP6, a mid-way introgression for which future selection of endosperm modification is required, the large residual region of opaque endosperm is contributing to reduced popping volume.

## Discussion

Popcorn enjoyed nearly a constant increase in sales during the second half of the twentieth century (Sweley et al., 2013) in part due to innovations such as microwave popcorn but sales have since plateaued. Strategies for invigorating the popcorn industry should be multipronged and involve agronomic improvement and other aspects such as nutritional quality. QPP, with its natural *opaque-2* and modifier alleles, and subsequent production of high yielding hybrids is one way improve the nutritional quality of popcorn and expand its realm. The development and utilization of more nutritious popcorn could have both economic and humanitarian application. Previous breeding focus on popping-related traits rather than plant agronomic traits, has resulted in much less agronomic improvement than in dent corn. For instance, yields of popcorn are less than dent corn (Ziegler, 2000). The agronomic superiority of dent corn has potential to contribute to popcorn improvements and led to several studies using crosses between dent corn and popcorn. Reduction of popping quality is commonly observed in dent corn by popcorn populations and thus is a major concern (Robbins and Ashman, 1984; Dofing et al., 1991; Lu et al., 2003; Li et al., 2006, 2009; Dhliwayo, 2008). However, studies confirmed that PEV could be partially recovered by backcrossing to popcorn parents while comparing the popping volume of F_1_, BC_1_, and BC_2_ populations (Crumbaker et al., 1949; Johnson and Eldredge, 1953). The complexity of the genetic loci contributing to PEV makes its maintenance challenging. Previously, it was reported that two generation backcrossing was sufficient to recover acceptable PEV (Crumbaker et al., 1949). The characteristic distinction in shape and size of kernels between popcorn and dent corn can be used for selection and accelerated recovery of popcorn genome. Using two generations of backcrossing combined with subsequent phenotypic selection for both kernel shape and ear characteristics, we selected inbred lines with comparable PEV to the original popcorn parents. Variation between BC_2_F_5_ populations indicated that there is scope for further selection of PEV. Besides recovery of popping, for introgression of dent traits into commercial lines with cross-incompatibility, maintaining *Ga1-s* is essential to maintain popcorn’s genetic isolation and ability to be grown in proximity to GM corn. We initiated F_1_ crosses unidirectionally using popcorn parental lines as males and then used the F_1_ and backcross generations as males to cross to popcorn females in subsequent generations. This ensured the maintenance of cross incompatibility between dent corn and popcorn since only pollen bearing the *Ga1-s* allele could successfully pollinate the popcorn recurrent parent.

As one way to avert protein malnutrition, many QPM conversion studies were carried out previously for dent corn (Babu et al., 2005; Gupta et al., 2009, 2013; Sofi et al., 2009; Jompuk et al., 2011; Krishna et al., 2017; Surender et al., 2017). Common challenges in QPM conversion programs included incomplete recovery of vitreous endosperm (modification), discontinuation of certain crosses from poor seed set and failure to improve lysine and tryptophan content (Moro et al., 1996; Prasanna et al., 2001; Vivek et al., 2008; Kostadinovic et al., 2016). Since popcorn kernels naturally have a very high proportion of vitreous endosperm (Figure 3), and this is the kernel region in which starch can melt during the popping process, complete endosperm modification is likely of paramount importance in QPM conversion into a popcorn background. *o2* mutants without modification are associated with soft kernels and loss of popping as observed in several *opaque-2* introgression studies (Zhou et al., 2016; Adunola, 2017). By evaluation of modifier transfer at the F_2_ generation, we targeted crosses at an early stage, that had greater potential for later full restoration of vitreous, and likely poppable kernels. For these promising crosses, selection for endosperm modification was carried out on an individual basis in BC_2_F_2_ generation (Type II and Type III opaque BC_2_F_2_ individuals) and then on a whole ear basis in later generations (BC_2_F_3_, BC_2_F_4_, BC_2_F_5_). Selection of Type II and Type III opaque kernels at the BC_2_F_2_ population is key step. The ongoing six BC_2_F_4_ QPP introgressions had, on average, four promising BC_2_F_2_ populations segregating for Type I through Type IV opaqueness whereas the discontinued crosses were the ones which showed close to 25% Type IV–V (unmodified/opaque) kernels segregating in BC_2_F_2_ populations.

Poor seed set has been observed in other QPM conversions throughout selection and can lead to the loss of targeted crosses (Kostadinovic et al., 2016). This phenomenon may be attributable to incompatibility between pollen and style. In our study, this was problematic in the BC_2_F_2_. Although many crosses seemed promising for modifier transfer with significant *p*-values (α = 0.05) in F_2_ populations, because of poor seed set in the BC_2_F_2_, we were not able to select enough Type II and Type III opaque BC_2_F_2_ individuals for some crosses (Supplementary Table S3). Also, failure to improve lysine content was reported in some QPM conversions despite successful introgression of *o2* and modifier genes (Moro et al., 1996; Babu et al., 2005; Vivek et al., 2008). However, this problem was not observed in any of our six BC_2_F_4_ introgressions and the increases in both protein-bound lysine and free lysine were significant for all BC_2_F_4_ QPP introgressions. RNA interference (RNAi) was adopted to reduce 22-kDa and 19-kDa zeins resulting in increased lysine (Wu and Messing, 2011). The process of proteome rebalancing means that decrease of lysine devoid zein proteins will result in increased accumulation of lysine containing non-zeins (Wu and Messing, 2014). In addition to this global increase in non-zeins, it was shown that the most elevated non-zein proteins are enriched in lysine (Morton et al., 2015). For QPP to be useful, it is important that the kernels do not have reduced total protein. No total protein reductions were observed compared with either the popcorn or QPM parents and in fact, a significant increase in total protein is shown in two of our introgressions (Supplementary Figure S12). Most free amino acids are increased in QPP (Supplementary Table S4B) and this may be partly as a result of reduced incorporation into zeins. Substantial increases in the levels of most free amino acids were observed in several types of maize mutants with reduced zeins (Galili and Amir, 2013). However, since zeins do not contain lysine, this does not explain the increase in free lysine. The free amino acids increase was initially demonstrated in the *o2* mutant. In addition to zein genes, *O2* also regulates other genes including one encoding a lysine catabolic enzyme lysine-ketoglutarate reductase/saccharopine dehydrogenase (LKR-SDH) (Brochetto-Braga et al., 1992; Kemper et al., 1999; Li et al., 2015). The resulting reduction in lysine degradation contributes to the observed free-lysine increase in *o2* mutant. PCA of protein-bound amino acids indicated that the QPP introgressions grouped with QPMs but were distant from the group of wild type popcorn parents. Amino acids showing uniform increase (Asx, His, Lys, Arg) or decrease (Met, Ser, Glx, Ala, Ile, Leu, Phe) between all *o2* (QPM parents, QPPs, and B73 *o2*) and all wild type germplasms (popcorn parents and B73) in this study were consistent with the comparison of amino acid composition between a wild type (W64A) and its isogenic line W64A*o2* (Hunter et al., 2002; Supplementary Figure S11). In the PCA analysis of free amino acids, wild type (popcorn parents, B73) were grouped together, whereas no obvious pattern were observed across different *o2* mutants (QPM parents, QPPs, and B73*o2*) (Supplementary Figure S10).

In our breeding project, because of the simultaneous initiation of many cross combinations, it was unfeasible to genotype all parental lines in terms of cost and time. To recover popcorn genome, we used a continuous two-time backcrossing strategy (theoretically 87.5% genome recovered for recurrent parent), which is cost-efficient and was reported to generate acceptable PEV (Crumbaker et al., 1949). With respect to our priority for endosperm modification (high percentage vitreous endosperm recovery, which is itself a major characteristic of popping potential) since generation of BC_2_F_2_, a direct evaluation of kernel vitreousness was carried out on the light box, without full knowledge of modifier QTLs for the QPM parents used. The increased uniformity in endosperm modification observed from BC_2_F_2_ to BC_2_F_4_ indicated that the modifiers likely reached homozygosity during this process. In BC_2_F_4_ populations tested with SDS-PAGE, the uniformly increased level of 27 kD γ-zein protein likely indicates that the allelic composition 27 kD γ-zein locus reached homozygosity for the duplicated allele. This process for recovery of endosperm modification would presumably have been longer, had we prioritized high percentage of popcorn parent genome along the breeding process. By combining backcrossing and phenotypic selection, generation advancement was carried out cost-effectively for multiple crosses simultaneously.

The strategy outlined here for introgression of dent corn traits into popcorn backgrounds brings together selection for QPM (*o2* and modifier genes) and for popcorn characteristics (cross-incompatibility conferred by Ga1-s, kernel shape, high proportion of vitreous endosperm), resulting in the first proof-of-concept demonstration that development of QPP is achievable. The six BC_2_F_4_ introgressions between three QPMs and four popcorn parents, all carrying the homozygous *o2* allele, give excellent scope for QPP hybrid production and testing which has begun.

## Data Availability

All data generated or analyzed during this study are included in this published article and its supplementary information files.

## Author Contributions

YR and DH designed the research. All authors performed the research. YR, AY, RA, and DH analyzed the data. YR and DH wrote the paper.

## Conflict of Interest Statement

The authors declare that the research was conducted in the absence of any commercial or financial relationships that could be construed as a potential conflict of interest.
