# Supplementary material for: Generation and Evaluation of Modified Opaque-2 Popcorn Suggests a Route to Quality Protein Popcorn
Source: Front Plant Sci. 2018 Dec 6;9:1803. doi: 10.3389/fpls.2018.01803 (PMC6291453; doi:10.3389/fpls.2018.01803)
Supplement: Supplementary file 1 [file Data_Sheet_1.pdf]

Supplementary Material

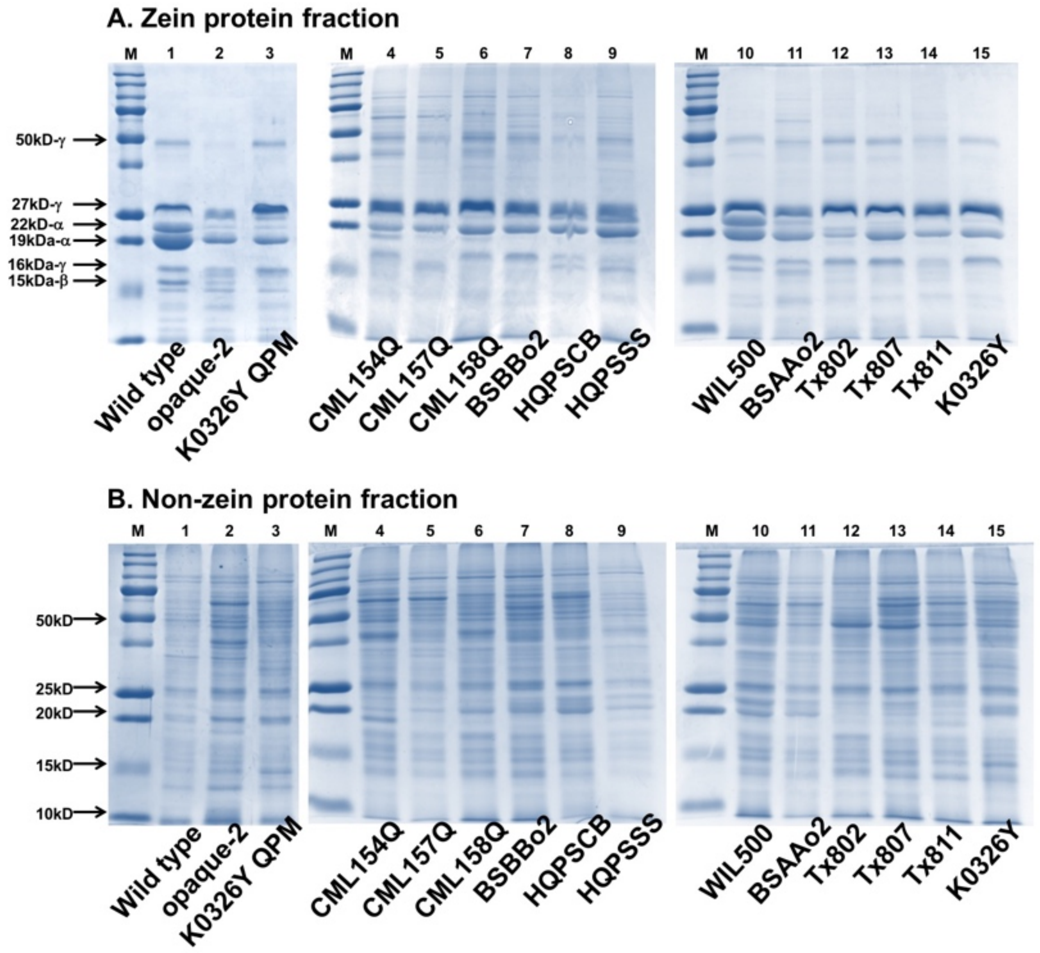

Fig. S1. SDS/PAGE analysis of zein proteins (A) and non-zein proteins (B) in wild type, *o2* mutant and 12 QPMs. (M: marker. 1. Wild type. 2. *opaque-2* mutant. 3. QPM K0326Y. 4. CML154Q. 5. CML157Q. 6. CML158Q. 7. BSBB*o2*. 8. HQPSCB. 9. HQPSSS. 10. WIL500. 11. BSAA*o2*. 12. Tx802. 13. Tx807 14. Tx811. 15. K0326Y). Compared with wild type (lane 1), all *o2* mutants except for HQPSSS (lane 9) and WIL500 (lane 10) have α-zein reduction. Compared with *opaque-2* mutant (lane 2), all 12 QPMs have increased level of 27kD γ-zein. Based on this analysis, two QPMs, WIL 500 and HQPSSS were not used as donor for *o2* and modifiers for later introgression.

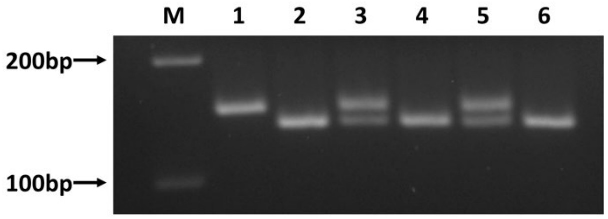

Fig. S2. Genotyping results for six randomly selected seedlings of QPM HQPSCB. (M. DNA marker. 1-6: six randomly selected individuals of HQPSCB.). Genotyping using marker umc1066 was carried out using DNA templates extracted from fresh leaf tissue of HQPSCB seedlings from six randomly selected kernels. This results showed that ear for HQPSCB was a mixture of kernels which were of homozygous genotype *O2O2* (lane 1), homozygous genotype *o2o2* (lanes 2,4,6) and heterozygous genotype *O2o2* (lanes 3, 5) for *opaque 2* locus.

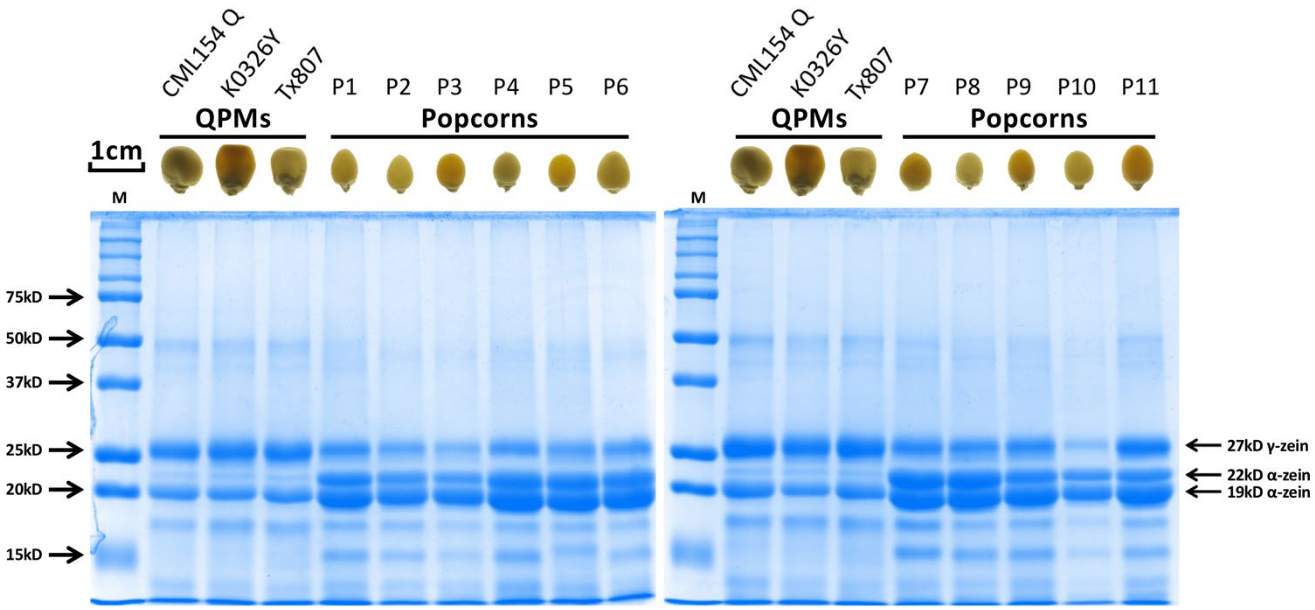

Fig. S3. Zein profile of three selected QPMs (CML154Q, K0326Y, Tx807) and 11 popcorn lines (P1-P11). All QPMs have reduced amount of 22kD α-zein and increased amount of 27kD γ-zein, consistent with the fact that they are modified *o2*. All popcorn kernels are wild type in that they don't have α-zein reduction. In terms of 27kD γ-zein, it seems that one popcorn line (P11) may have increased amount of 27 kD γ-zein, which may facilitate endosperm modification.

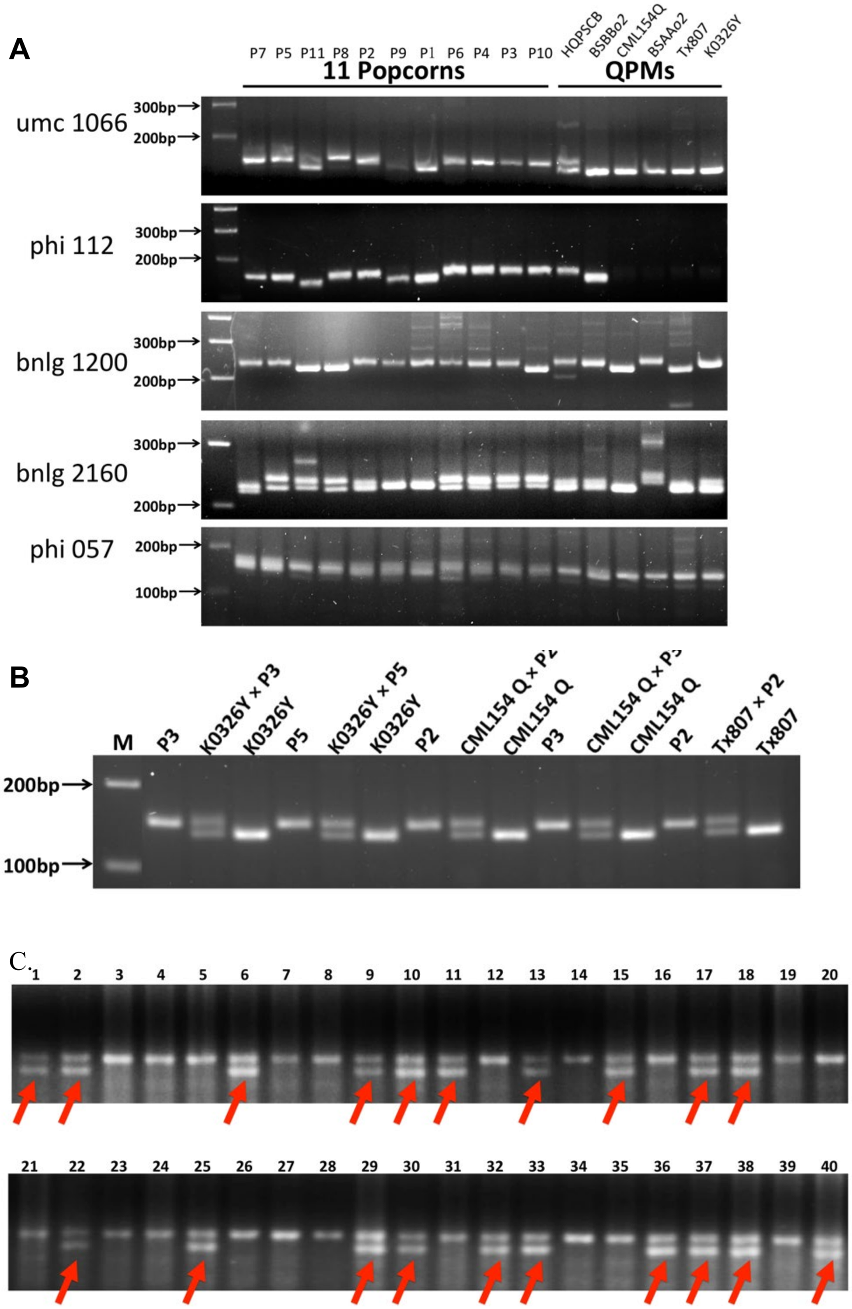

Fig. S4. Screening of five pairs of *o2* markers (umc1066, phi112, bnlg1200, bnlg2160, phi057) across 11 popcorn lines and six QPMs (A), umc1066 is a co-dominant marker (B) and application of umc1066 in BC_1 and BC_2 generations for cross K0326Y x P3 (C). In A, in total, six QPMs were included because, F_1 were successfully made using these six QPMs as female parents (Table S1.). umc1066 was selected for foreground selection of *opaque 2* because it gave positive amplification for all lines, showed most polymorphism and is a co-dominant marker indicating all three genotypes (*O2O2*, *O2o2*, *o2o2*). In B, the polymorphisms for F_1 crosses (K0326Y x P3, K0326Y x P5, CML154 Q x P2, CML154Q x P3, Tx807 x P2) with both parents by the sides confirmed that umc1066 is a co-dominant marker which is useful to differentiate genotypes (*O2O2*, *O2o2*, *o2o2*). In C, individuals in BC_1 (up, lane 1-20) and BC_2 (bottom, lane 21-40) between QPM K0326Y and P3 were genotyped using umc1066, heterozygous genotypes (*O2o2*) carrying the *opaque 2* mutant allele were pointed out with red arrows.

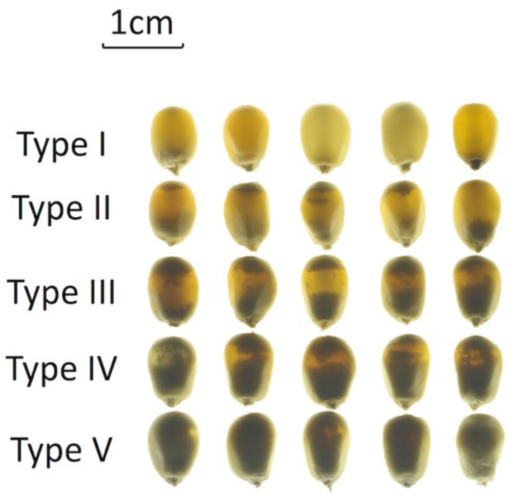

Fig. S5. Classification of endosperm vitreousness of F_2 population (K0326Y x P2) where Type I is fully vitreous and Type V is fully opaque. This classification facilitated evaluation of modifier transfer in F_2 populations. It was also applied to BC_2F_2 populations for selection of Type II and Type III opaque kernels which were confirmed to be *o2* mutants.

A

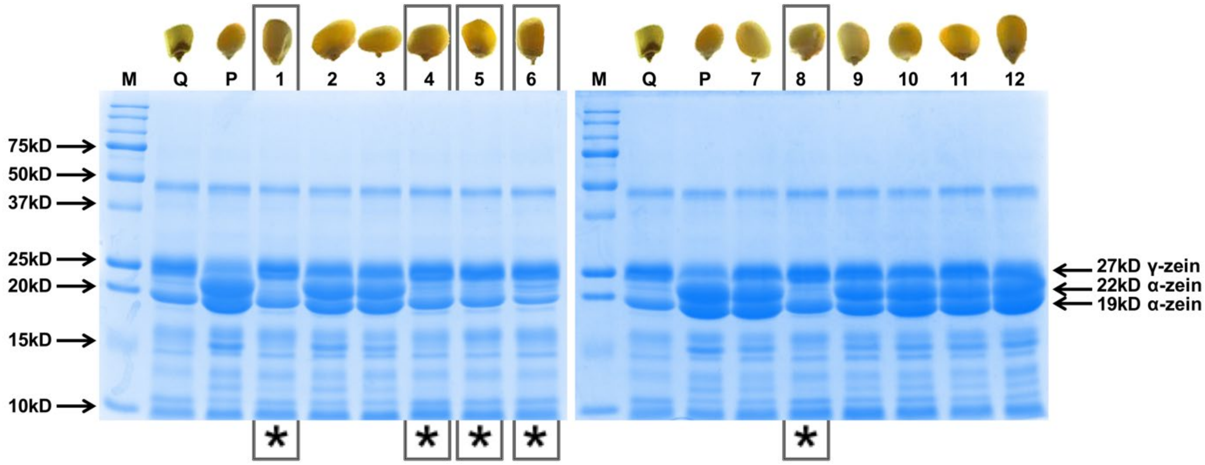

B

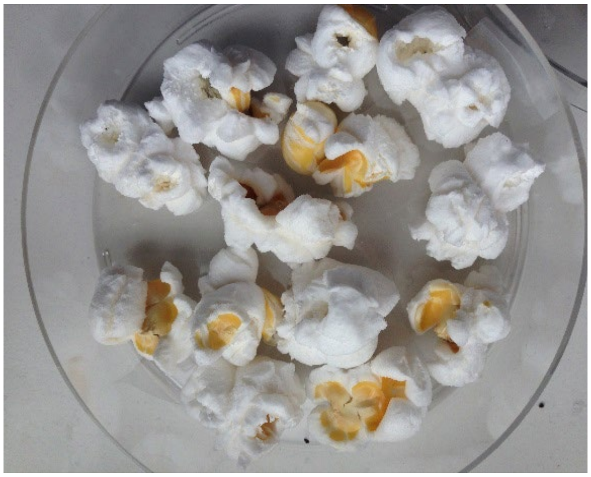

Fig. S6. Identification of completely modified poppable *o2* mutants in F_2 population from cross Tx807 x P7. In A, Q. Tx807, P, popcorn parent P7. 1-12 were kernels randomly selected from a pool of roundish fully vitreous kernels in F_2 population from cross Tx807 x P7. Boxes and asterisks represent vitreous popcorn-like kernels, which were QPM since they have low α-zein and high 27kD γ-zein. In B, fifteen kernels selected from the same pool of roundish fully vitreous kernels were used for preliminary popping. Although kernels used for popping are not the same kernels as in A tested for zein profile, these are by definition ~25% *o2o2*.

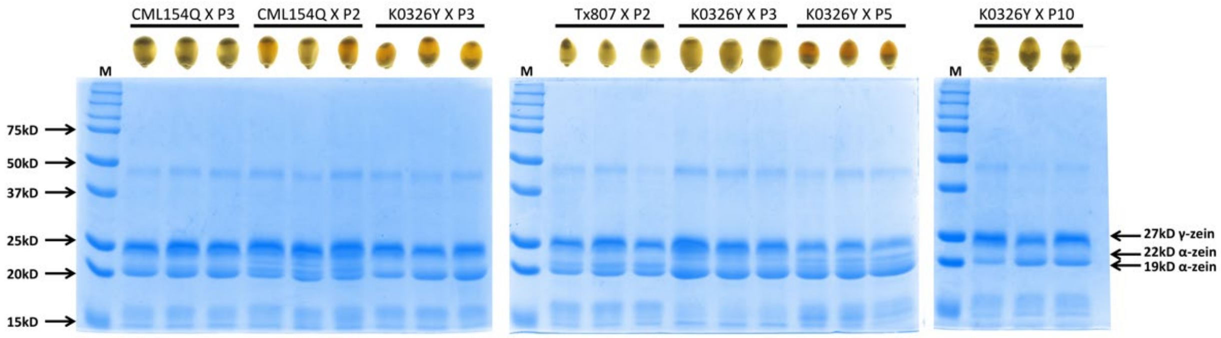

Fig. S7. Confirmation of Type II and Type III opaque kernels in BC_2F_2 generation as *o2* mutants with low α-zein, high γ-zein compared to corresponding popcorn parental lines in Figure. S3. Type II and Type III opaque kernels in BC_2F_2 generation were selected for generation advancements. Three such kernels were randomly selected from each ear to test zein profile to see if they are *o2* and the seven crosses tested here are CML154Q x P3, CML154Q x P2, K0326Y x P3, Tx807 x P2, K0326Y x P3, K0326Y x P5, K0326Y x P10. From the zein protein profile, these kernels were confirmed to be *o2* mutants because of reduction of 22kDα-zein, they have increased 27 kD γ-zein which is consistent with the fact that these kernels are modified.

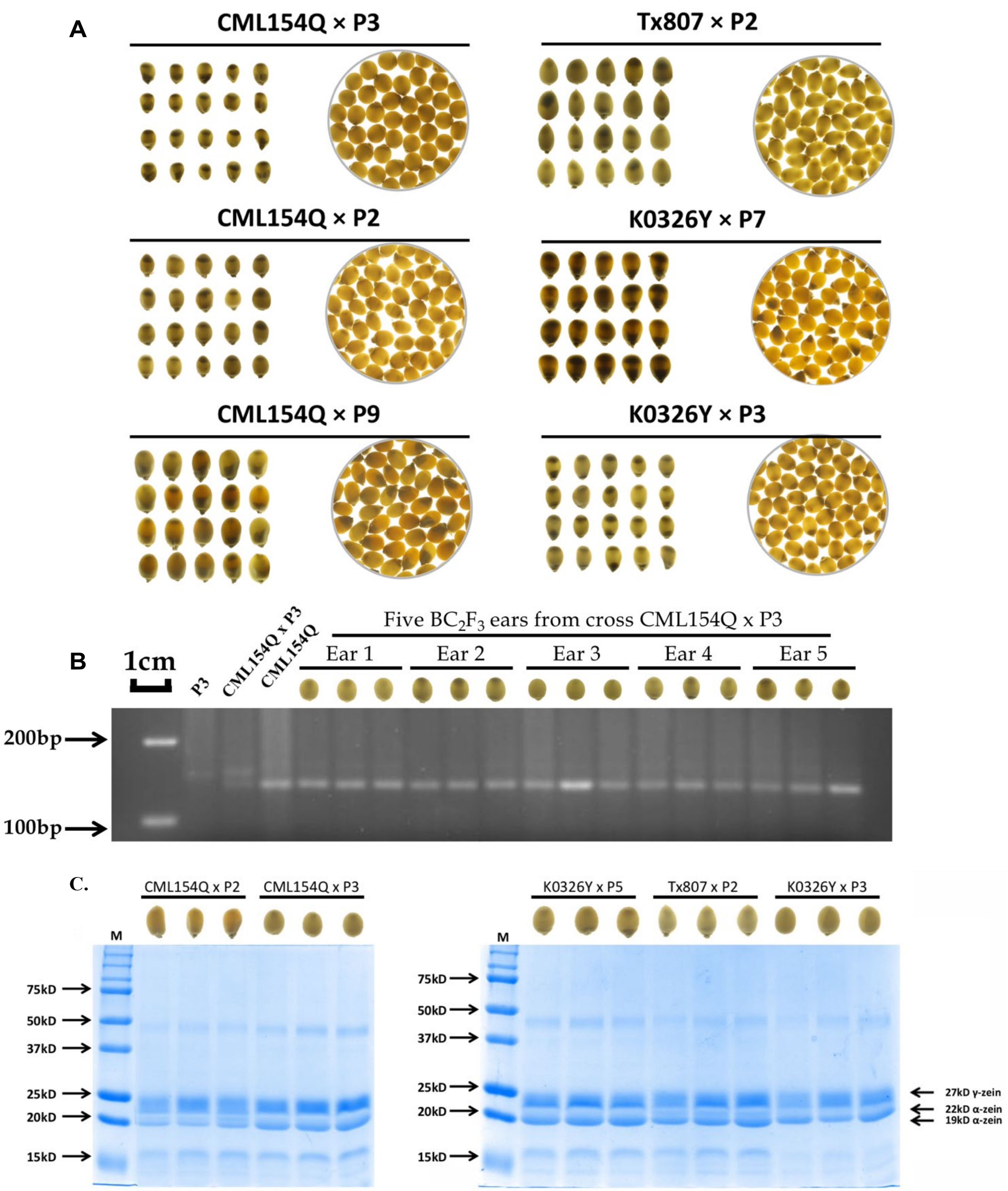

Fig. S8. Presence of Type I (vitreous) *o2* mutants in BC_2F_3 populations from partially modified (Type II and Type III opaqueness) BC_2F_2 individuals. A. Self-pollinating partially modified *o2* mutants selected from BC_2F_2 generation can give rise to BC_2F_3 population with increased modification. Examples from six introgressions which were CML154Q x P3, CML154Q x P2, CML154Q x P9, Tx807 x P2, K0326Y x P7, K0326Y x P3 were included here. For each of these introgressions, 20 Type II and Type III opaque BC_2F_2 kernels selected for planting in 2016 summer were shown on the left. After self-pollination, light box phenotyping was carried out on resultant BC_2F_3 populations. To compare endosperm modification with Type II and Type III BC_2F_2 individuals, best modified BC_2F_3 ear for each cross was selected and fully vitreous kernels from this ear were shown on the right (right, in circles) for each introgression. Same selection was applied to introgression from F_1 cross K0326Y x P5, which were not included in this figure because of only 10 kernels in BC_2F_2 population were selected as Type II and Type III opaque kernels. B. Confirmation of vitreous BC_2F_3 individuals to be *o2* mutants. For cross CML154Q x P3, five promising ears were selected in BC_2F_3 generation. To confirm that these kernels with increased modification were *o2* mutants, three such kernels were randomly selected from each ear. The results showed that all these kernels are *o2* mutant. C. Confirmation of vitreous kernels in the BC_2F_3 generation to be *o2* mutants with reduced α-zein and increased 27kD γ-zein to guarantee modification. Three kernels with Type I opaqueness were selected from each promising BC_2F_3 population. There seemed to be variation between individuals in terms of 27kD gamma zein expression.

A

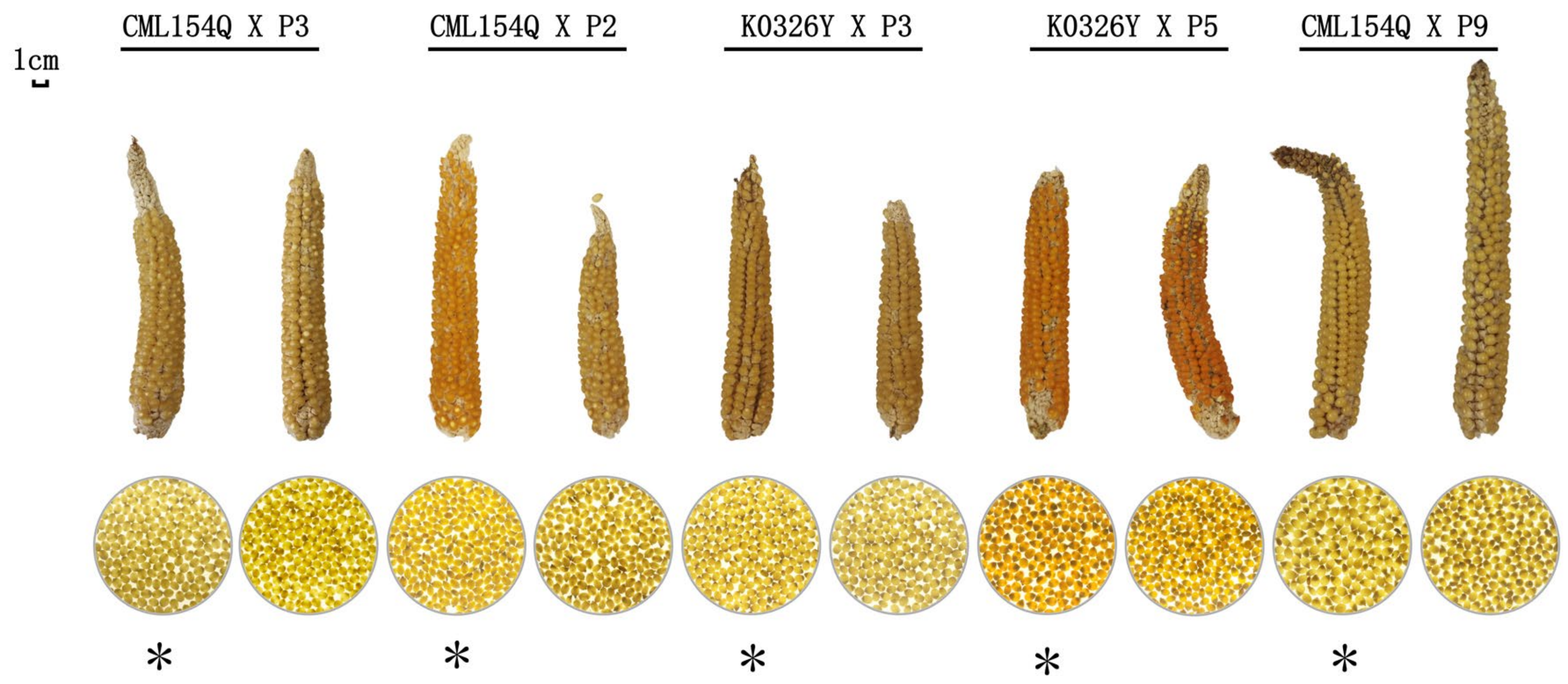

Fig. S9. Kernel and zein phenotype of BC_2F_4 populations. A. Promising BC_2F_4 introgressions with uniform modification. In total, two ears were selected for each of the five introgressions, which were originated from crosses CML154Q x P3, CML154Q x P2, K0326Y x P3, K0326Y x P5 and CML154Q x P9 respectively. Tx807 introgression (originated from Tx807 x P2) was not included because the ear was not fully filled. Compared with the other five introgressions in kernel modification, this cross was a little behind in that the kernels were between Type I and Type II opaqueness (Fig.3). For Tx807 introgression, BC_2F_4 individuals were prepared for amino acid profiling analysis, the rest were self-pollinated to restore modification. BC_2F_5 populations for this cross were included in quantitative popping analysis. For each of the five crosses, two independent introgressions were carried onward with. The ones marked with asterisks were selected for amino acid profiling analysis

B

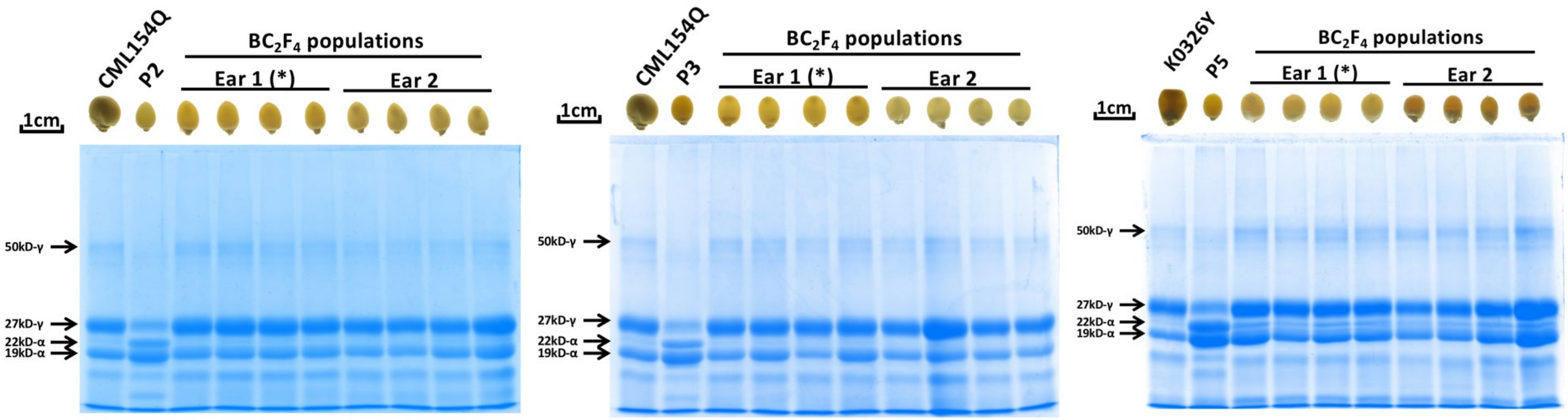

Fig. S9. (Continued) B. SDS-PAGE confirmation of *o2* mutants in vitreous BC_2F_4 populations. Besides the two introgressions originated respectively from crosses CML154Q x P9 and K0326Y x P3 shown in Fig.2, confirmation of completely modified *o2* mutants for introgressions from cross CML154Q x P2 (left), CML154Q x P3 (middle) and K0326Y x P5 (right) are shown here.

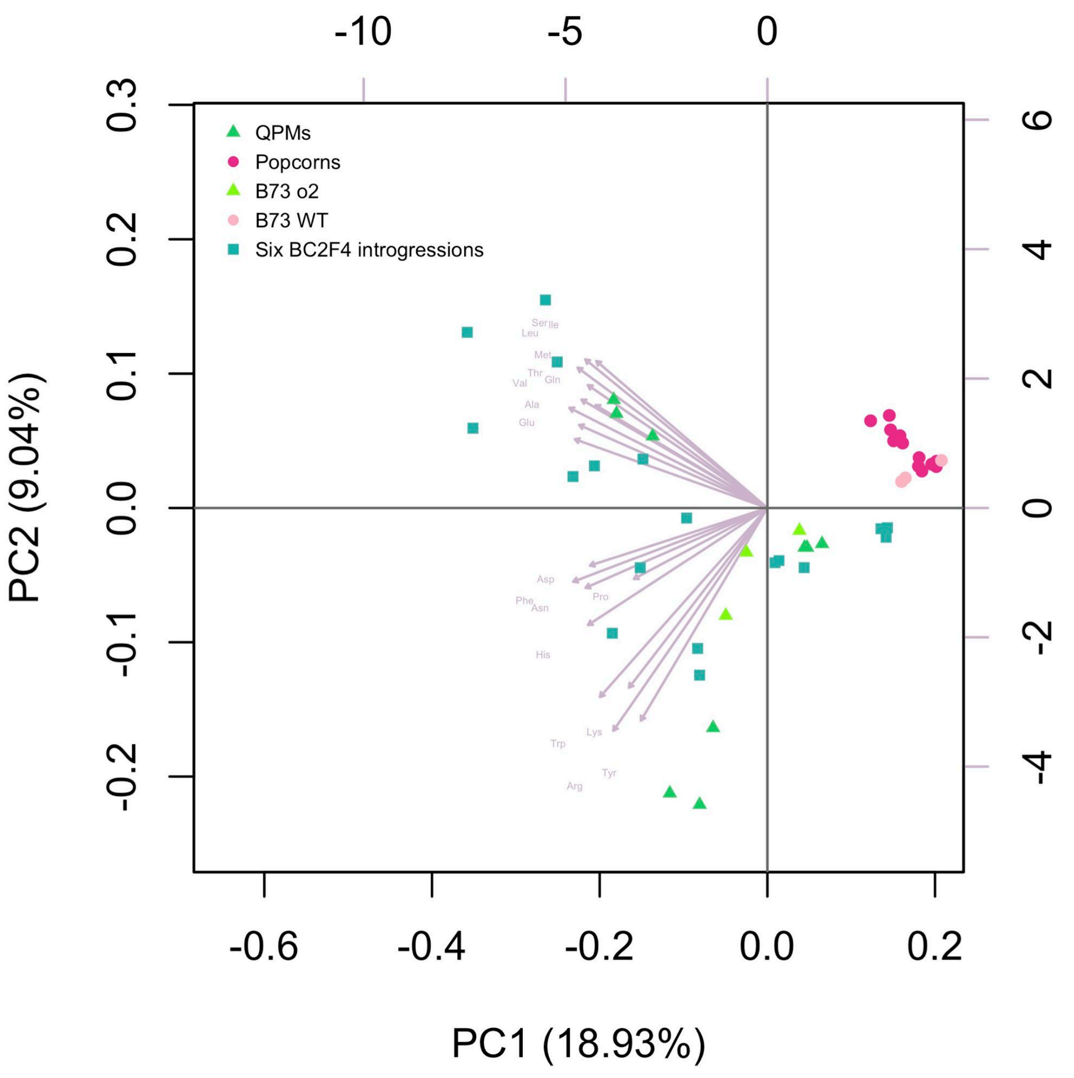

Fig. S10. PCA biplot for QPM, popcorn and six BC_2F_4 introgressions using free amino acids measurements. PCA of free amino acid data showed that all wild type germplasms grouped together. Three QPMs were distant with the wild type group. This shows variation between three QPMs and no obvious pattern for *o2* germplasms in terms of free amino acids.

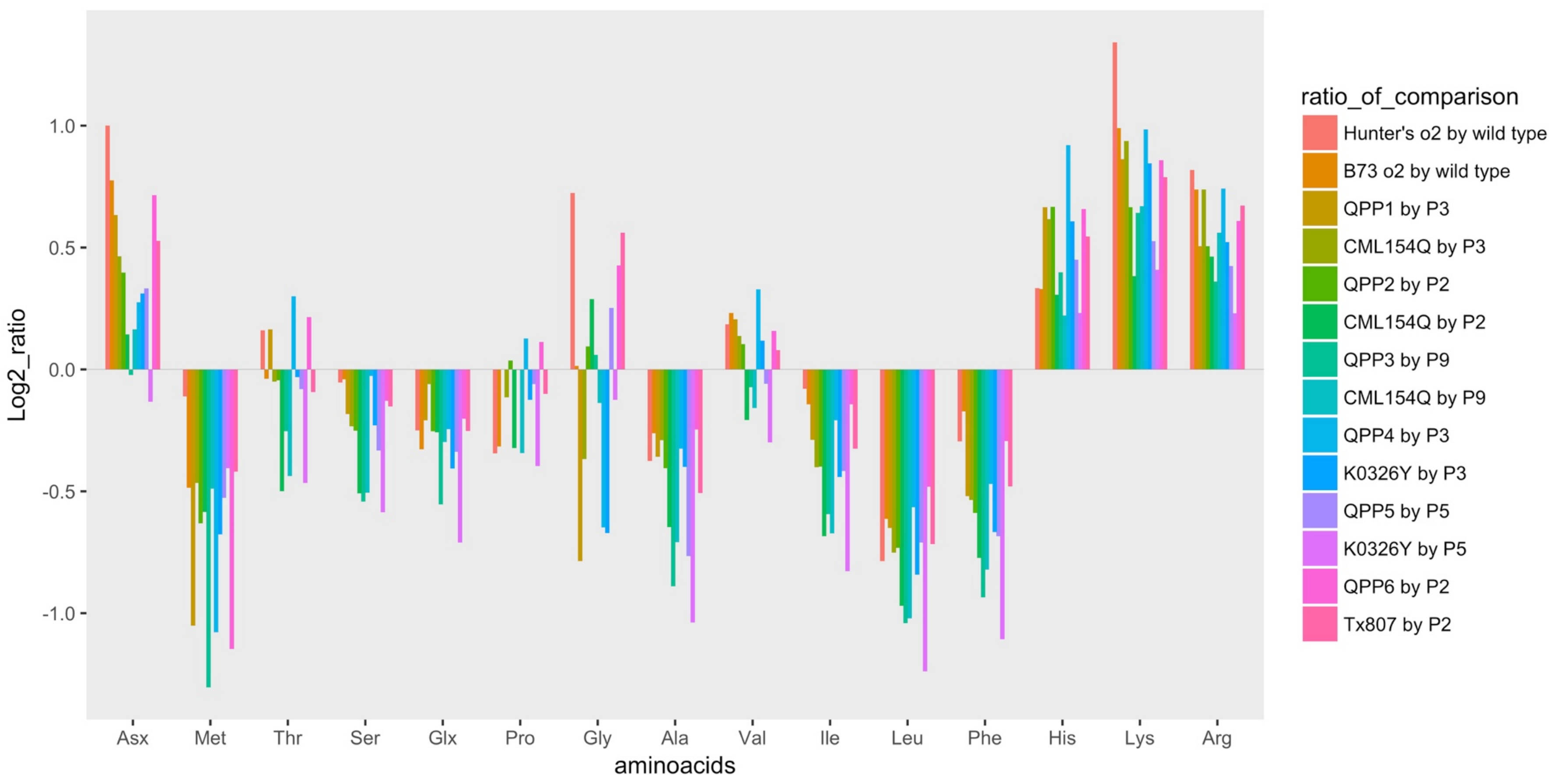

Fig. S11. Comparison of log2 value of ratio (*o2* / wild type ratio) for 15 amino acids using protein bound amino acid measurements. Horizontal axis showed the 15 amino acids shared commonly between our protein bound amino acid data and Hunter's data which are respectively Asx (both aspartic acid and asparagine), Met, Thr, Ser, Glx (both glutamic acid and glutamine), Pro, Gly, Ala, Val, Ile, Leu, Phe, His, Lys and Arg. The 14 bars in each of the fifteen amino acids represent the log 2 value of the ratio for the amount of certain amino acid between *o2* and wild type in Hunter's data, B73*o2* and B73 wild type, QPP1 (introgression from cross CML154Q x P3) and P3, CML154Q and P3, QPP2 (introgression from cross CML154Q x P2) and P2, CML154Q and P2, QPP3 (introgression from cross CML154Q x P9), CML154Q by P9, QPP4 (introgression from cross K0326Y x P3) and P3, K0326Y x P3, QPP5 (introgression from cross K0326Y x P5) and P5, K0326Y x P5, QPP6 (introgression from cross Tx807 x P2) and P2, Tx807 x P2. Amino acids (Met, Leu, Phe, His, Lys, Arg) with uniform pattern (with log2 of the ratio being all positive or negative) across 14 comparisons implies their contribution to *o2* and wild type difference in general and lysine is one of them. The fact that 11 out of 15 amino acids showed uniform pattern across all 14 comparisons implies the change of proteins is similar across *o2* and wild type germplasms.

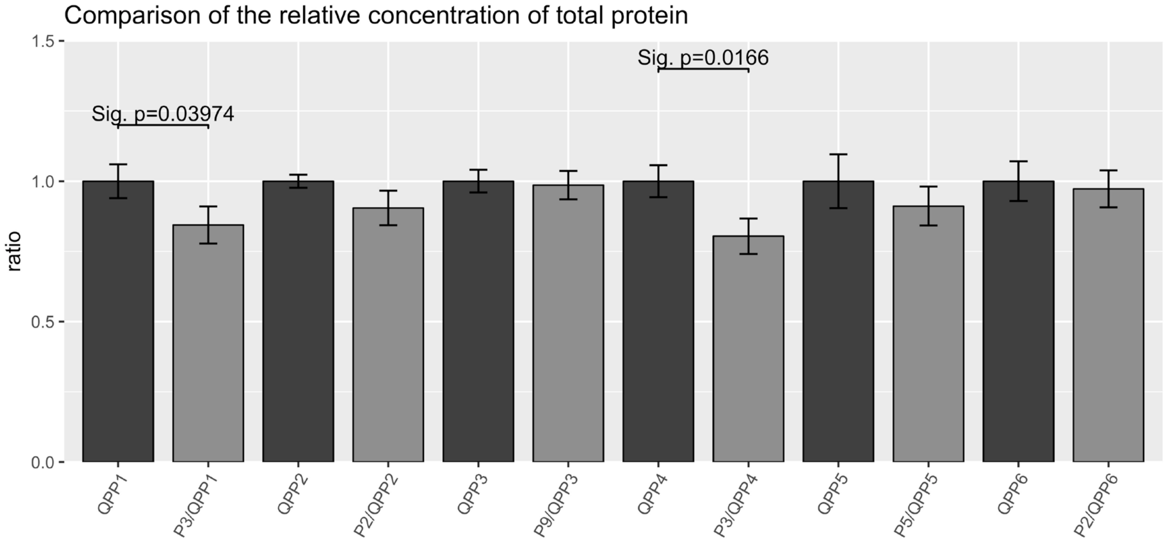

Fig. S12. Comparison of relative concentration for total protein between BC_2F_4 QPP introgressions and corresponding popcorn parental lines. Average of total protein in six QPPs were considered as 1. Relatively, the amount of total protein in corresponding popcorn parental lines were indicated as ratio to corresponding QPPs. For QPP1, the increase in total protein is significant when compared with corresponding popcorn parent P3 (p=0.03974). Significant increase was found in QPP4 compared with corresponding popcorn parent P3 (p=0.0166) (α=0.05).

Table S1. Characteristics of the 12 QPMs originally considered

pedigree	Source	Vitreous/ Opaque	Kerne l color	F1 made	Relative time to flowering	Zein content	Lysine
CML154Q	Ames27083	Mostly vitreous, smaller, roundish	white	Yes	2 weeks late	High γ, Low α	0.37±0.06
CML157Q	Ames27084	Mostly vitreous	white	No	3 weeks late, no pollination	High γ, Low α	NA
CML158Q	Ames27085	All fully vitreous	white	No	4 weeks late, no pollination	High γ, Low α	NA
BsBB*o2*(S)C 2	PI550495	Mix of vitreous, semi-vitreous, opaque	yellow	Yes	Normal	High γ, Low α	0.45±0.06
HQPSCB	PI586689	Fully vitreous, large	yellow	Yes	1-2 weeks late	High γ, Low α	0.35±0.06
HQPSSS	PI586690	Fully vitreous, large	yellow	Yes	Normal	High γ, High α	0.34±0.04
WIL500	PI601689	Vitreous, large, roundish	yellow	Yes	2 weeks late	High γ, High α	0.36±0.02
BSAA*o2*(S)C1	PI608781	Mostly opaque	yellow	Yes	Normal	High γ, Low α	0.44±0.06
Tx802	PI619429	All modified, smaller kernel	yellow	No	2 weeks late	High γ, Low α	0.42±0.02
Tx807	PI619430	All super vitreous, smaller kernel	white	Yes	2 weeks late	High γ, Low α	0.45±0.01
Tx811	PI619431	Mix of vitreous and semi-vitreous	white	No	3 weeks late, no pollination	High γ, Low α	NA
K0326Y	A tropical QPM inbred developed by Hans Geevers	All vitreous, roundish	yellow	Yes	3 weeks late, no pollination	High γ, Low α	0.39±0.01

Note. Highlighted in yellow are the QPM parents for ongoing QPP introgressions. Amino acid quantification was carried out by Agricultural Experiment Station Chemical Laboratories in University of Missouri-Columbia in 2014. For lysine measurement, NA = 'not measured' (for CML157Q, CML158Q, Tx811). For these three QPM lines, no cross was made because of difference in time to flowering. Lysine measurements for comparison: lysine measurement for unmodified *o2* mutant B73*o2* is 0.60±0.02. Lysine measurement for B73 wildtype is 0.32±0.01.

Table S2. Primers screened for polymorphism across three QPMs and 11 popcorn lines

Prime Name	Sequence (5' to 3')	Note on application
phi057F	CTCATCAGTGCCGTCGTCCAT	*o2* in-gene marker
phi057R	CAGTCGCAAGAAACCGTTGCC	*o2* in-gene marker
umc1066F	ATGGAGCACGTCATCTCAATGG	*o2* in-gene marker
umc1066R	AGCAGCAGCAACGTCTATGACACT	*o2* in-gene marker
phi112F	TGCCCTGCAGGTTCACATTGAGT	*o2* in-gene marker
phi112R	AGGAGTACGCTTGGATGCTCTTC	*o2* in-gene marker
bnlg2160F	GAAGCAACCCATTTTCATCC	*o2* flanking marker
bnlg2160R	AGATTGGATTCCTGCCTCCT	*o2* flanking marker
bnlg1200F	CGTCCTCGTTGTTATTCCGT	*o2* flanking marker
bnlg1200R	GTTCCCTCTCTCCCTCCCTC	*o2* flanking marker

Table S3. Evaluation of modifier transfer at F_2 stage

Germplasm	Number of observed opaque kernels (Type III ~ Type V)	Number of observed vitreous kernels	p-value	Chi-square test χ2
**CML154 x P2**	**178**	**1695**	**2.20E-16***	**239.89**
**CML154Q x P3**	**147**	**1069**	**2.20E-16***	**108.11**
**CML154Q x P9**	**231**	**1471**	**2.20E-16***	**118.54**
K0326Y x P6	273	854	0.5472	0.36232
K0326Y x P4	227	714	0.5345	0.38576
K0326Y x P10	308	1058	0.03633*	4.3816
K0326Y x P7	181	872	4.81E-09*	34.264
**K0326Y x P5**	**277**	**922**	**0.1292**	**2.3022**
**K0326Y x P3**	**188**	**806**	**9.35E-06***	**19.639**
**Tx807 x P2**	**281**	**1050**	**0.001054***	**10.731**
Tx807 x P7	220	902	3.03E-05*	17.399

Note. Three F_2 ears were randomly selected for each cross. Number of opaque kernels (Type III ~Type V) and number of vitreous kernels were summed up respectively from three ears. Theoretically, it there is no modifier, *opaque2* would be segregating 1:2:1 (*o2/o2* : *O2/o2* : *O2/O2*) genotypically resulting phenotypically segregating for endosperm modification with a ratio of 1:3 (opaque : vitreous). Deviation of the ratio for endosperm modification from 1:3 reflects the effect of modifier in the F_2 stage. Chi-Square analysis was carried out for pools of three F_2 populations for 11 crosses. The result was summarized in Table S3. Significant p–values (α=0.05) was indicated by asterisk (*). Crosses with advanced BC_2F_4 introgressions were highlighted in bold. For cross K0326Y x P10, only five BC_2F_2 ears were harvested to select from and none gave enough kernels with Type II and Type III modification. For cross K0326 Y x P7, one promising BC_2F_2 ear was selected out of 14 ears but selection stopped at BC_2F_3 stage because there were no signs for improved modification. For cross Tx807 x P7, six ears were harvested and none of them showing acceptable modification. For four of the six QPP introgressions in our study which were from cross CML154Q x P2, CML154Q x P3, K0326Yx P3 and CML154Q x P9, at least four BC_2F_2 ears segregating for endosperm modification were received for selection of Type II and Type III kernels from. Three such BC_2F_2 ears were selected for QPP6 (from cross Tx807 x P2). Two such BC_2F_2 ears were selected for QPP5 (from cross K0326Y x P5).

Table S4. Amino acid profile of QPMs, popcorn parents, and six different introgressions (BC_2F_4)

A. Protein-bound amino acids.

AA[a]	CML154Q	K0326Y	Tx807	P3	P2	P5	P9	QPP1	QPP2	QPP3	QPP4	QPP5	QPP6	B73 wt	B73 *o2*
Ala	0.55±0.03	0.51±0.01	0.60±0.04	0.67±0.02	0.85±0.10	1.04±0.10	0.89±0.05	0.52±0.07	0.65±0.01	0.48±0.02	0.53±0.02	0.61±0.06	0.72±0.05	0.96±0.04	0.80±0.11
Arg	0.29±0.04	0.25±0.02	0.34±0.02	0.18±0.01	0.21±0.01	0.21±0.01	0.20±0.02	0.25±0.00	0.30±0.01	0.25±0.00	0.29±0.01	0.29±0.01	0.32±0.01	0.45±0.05	0.76±0.08
Asx	0.79±0.02	0.72±0.04	1.04±0.09	0.58±0.03	0.72±0.01	0.78±0.01	0.71±0.07	0.89±0.03	0.95±0.04	0.70±0.03	0.70±0.02	0.99±0.07	1.18±0.03	0.69±0.04	1.18±0.07
Glx	1.40±0.38	1.10±0.07	1.40±0.09	1.46±0.05	1.67±0.08	1.80±0.03	1.72±0.20	1.26±005	1.40±0.06	1.17±0.00	1.23±0.04	1.42±0.04	1.45±0.01	2.21±0.11	1.76±0.25
Gly	0.57±0.11	0.46±0.01	0.69±0.14	0.74±0.53	0.47±0.11	0.51±0.09	0.63±0.16	0.43±0.08	0.50±0.13	0.66±0.17	0.47±0.08	0.60±0.17	0.63±0.25	0.84±0.08	0.85±0.03
His	0.31±0.01	0.31±0.01	0.37±0.01	0.20±0.01	0.25±0.01	0.26±0.01	0.27±0.03	0.32±0.02	0.40±0.01	0.35±0.01	0.38±0.01	0.36±0.01	0.40±0.02	0.27±0.01	0.34±0.03
Ile	0.39±0.01	0.38±0.01	0.50±0.02	0.51±0.02	0.63±0.03	0.67±0.00	0.62±0.06	0.42±0.03	0.47±0.01	0.41±0.01	0.45±0.02	0.50±0.02	0.57±0.02	0.48±0.03	0.44±0.07
Leu	0.89±0.06	0.83±0.03	1.06±0.08	1.50±0.03	1.74±0.06	1.97±0.01	1.80±0.22	0.95±0.10	1.05±0.08	0.88±0.04	1.01±0.04	1.20±0.02	1.25±0.06	1.50±0.08	0.98±0.16
Lys	0.31±0.01	0.29±0.02	0.41±0.04	0.16±0.02	0.24±0.01	0.22±0.01	0.19±0.02	0.29±0.00	0.38±0.02	0.30±0.01	0.32±0.01	0.32±0.02	0.43±0.01	0.24±0.02	0.48±0.04
Met	0.07±0.02	0.06±0.01	0.07±0.01	0.09±0.02	0.10±0.00	0.07±0.02	0.09±0.00	0.04±0.00	0.06±0.01	0.04±0.01	0.04±0.00	0.05±0.00	0.04±0.01	0.26±0.01	0.19±0.02
Phe	0.36±0.02	0.32±0.01	0.44±0.02	0.52±0.03	0.61±0.05	0.70±0.02	0.63±0.08	0.36±0.03	0.40±0.02	0.33±0.01	0.37±0.02	0.43±0.02	0.50±0.03	0.56±0.05	0.50±0.11
Pro	0.83±0.04	0.82±0.02	0.96±0.02	0.89±0.05	1.03±0.03	1.08±0.04	1.05±0.11	0.89±0.06	1.06±0.06	1.04±0.06	0.98±0.04	1.03±0.05	1.12±0.03	0.85±0.04	0.69±0.07
Ser	0.40±0.02	0.40±0.01	0.51±0.02	0.47±0.03	0.57±0.00	0.61±0.01	0.57±0.07	0.42±0.02	0.48±0.01	0.39±0.01	0.46±0.01	0.48±0.02	0.52±0.03	0.56±0.03	0.55±0.06
Thr	0.32±0.02	0.32±0.03	0.42±0.03	0.33±0.02	0.45±0.05	0.45±0.03	0.43±0.07	0.37±0.03	0.44±0.02	0.36±0.03	0.41±0.02	0.42±0.02	0.53±0.03	0.54±0.02	0.52±0.06
Tyr	0.18±0.06	0.18±0.01	0.24±0.02	0.27±0.00	0.32±0.00	0.33±0.01	0.31±0.04	0.19±0.01	0.23±0.02	0.19±0.01	0.20±0.01	0.23±0.00	0.25±0.01	0.27±0.01	0.21±0.02
Val	0.51±0.01	0.50±0.02	0.62±0.01	0.46±0.02	0.59±0.02	0.62±0.01	0.57±0.06	0.53±0.04	0.63±0.02	0.54±0.00	0.58±0.00	0.59±0.02	0.65±0.02	0.54±0.02	0.64±0.06

Note: AA[a], protein bound amino acids of sixteen amino acids (g/100g flour). The acid hydrolysis method used lead to the destruction of tryptophan which is therefore not detectable. Amino acids content refers to mean ± standard deviation (n=3). B73 wt and B73 *o2* were included for comparison.

Table S4. Continued

B. Free amino acids

AA[b]	CML154Q	K0326Y	Tx807	P3	P2	P5	P9	QPP1	QPP2	QPP3	QPP4	QPP5	QPP6	B73 wt	B73 o2
**Ala**	1.44E-02± 1.47E-03	7.29E-03± 7.31E-04	3.60E-02± 7.47E-03	2.21E-03± 1.45E-04	3.10E-03± 3.61E-04	1.15E-02± 1.88E-03	6.77E-03± 2.31E-03	2.38E-02± 4.79E-03	4.35E-02± 2.43E-02	6.69E-03± 3.73E-04	5.37E-03± 1.02E-03	4.49E-02± 5.89E-02	1.85E-02± 2.52E-03	7.71E-03± 3.99E-03	2.49E-02± 5.30E-03
**Arg**	4.23E-02± 3.43E-03	1.63E-02± 1.78E-03	1.49E-02± 3.10E-03	2.82E-03± 5.20E-04	3.76E-03± 3.79E-04	2.14E-03± 8.09E-05	1.75E-03± 4.42E-04	1.52E-02± 2.58E-03	2.16E-02± 5.17E-03	1.13E-02± 1.16E-03	1.16E-02± 1.67E-03	3.18E-02± 6.04E-02	3.76E-02± 3.80E-03	5.00E-03± 1.98E-03	1.63E-02± 7.20E-03
**Asn**	1.54E-01± 1.86E-02	1.01E-01± 7.63E-03	1.67E-01± 2.78E-02	1.79E-02± 2.17E-03	1.99E-02± 3.81E-03	1.78E-02± 3.92E-03	3.43E-02± 1.29E-02	2.03E-01± 2.87E-02	1.15E-01± 9.58E-03	9.01E-02± 9.91E-03	7.80E02± 1.30E-02	2.25E-01± 6.21E-02	2.52E-01± 1.56E-02	1.45E-02± 5.66E-03	4.88E-02± 1.87E-03
**Asp**	7.59E-02± 7.46E-03	7.45E-02± 8.42E-03	9.10E-02± 7.11E-03	4.89E-03± 7.36E-04	5.95E-03± 3.39E-03	1.05E-02± 9.86E-04	1.31E-02± 4.14E-03	1.47E-01± 1.41E-02	1.55E-01± 3.93E-03	7.06E-02± 9.70E-04	2.94E-02± 5.01E-03	1.05E-01± 3.96E-02	1.25E-01± 7.05E-03	1.05E-02± 3.53E-03	1.66E-01± 3.24E-02
**Gln**	3.83E-03± 2.79E-03	7.43E-04± 7.46E-05	1.52E-02± 8.85E-03	1.04E-03± 2.89E-04	1.05E-03± 3.44E-04	3.27E-03± 5.42E-04	3.84E-03± 1.79E-03	5.88E-02± 1.94E-02	4.77E-02± 2.71E-02	1.83E-02± 4.39E-03	1.50E-03± 2.06E-04	2.79E-02± 3.49E-02	3.29E-02± 1.68E-02	2.20E-03± 1.19E-03	6.83E-03± 4.14E-04
**Glu**	2.89E-02± 1.17E-02	2.07E-02± 2.37E-03	9.39E-02± 3.58E-02	9.03E-03± 1.43E-03	1.41E-02± 5.22E-04	9.52E-03± 1.29E-03	2.03E-02± 4.77E-03	1.55E-01± 8.66E-03	1.33E-01± 4.11E-02	8.04E-02± 1.38E-02	1.85E-02± 1.68E-03	1.18E-01± 3.59E-02	1.04E-01± 2.28E-02	1.94E-02± 9.54E-03	5.32E-02± 5.20E-03
**His**	6.33E-03± 8.04E-04	5.69E-03± 3.19E-04	6.30E-03± 8.54E-04	1.06E-03± 1.94E-04	1.68E-03± 1.46E-05	1.72E-03± 6.18E-05	1.20E-03± 9.28E-05	4.14E-03± 2.81E-04	7.18E-03± 1.50E-03	3.07E-03± 1.83E-04	3.54E-03± 3.83E-04	9.65E-03± 2.33E-02	9.45E-03± 4.49E-04	1.28E-03± 3.50E-04	5.79E-03± 1.31E-03
**Ile**	5.54E-04± 1.50E-04	8.10E-04± 6.38E-05	2.61E-03± 2.99E-04	2.37E-04± 8.74E-05	2.63E-04± 9.18E-05	5.50E-04± 5.45E-05	7.08E-04± 8.04E-05	1.70E-03± 5.04E-04	1.29E-03± 5.67E-04	5.93E-04± 1.73E-04	3.00E-04± 7.90E-06	2.14E-03± 2.43E-02	4.91E-04± 8.92E-05	3.77E-04± 7.01E-05	1.03E-03 ±1.05E-04
**Leu**	9.21E-04± 2.14E-04	9.53E-04± 6.08E-05	2.67E-03± 1.23E-04	2.83E-04± 9.51E-06	3.15E-04± 2.74E-06	5.97E-04± 1.04E-04	7.08E-04± 8.04E-05	3.50E-03± 8.23E-04	3.39E-03± 1.87E-03	7.85E-04± 8.35E-05	3.33E-04± 5.86E-05	2.49E-03± 2.52E-02	1.17E-03± 6.73E-04	4.26E-04± 1.55E-05	9.26E-04± 1.54E-04
**Lys**	1.53E-02± 2.19E-03	9.39E-03± 8.66E-04	1.39E-02± 1.96E-03	1.47E-03± 2.77E-04	2.28E-03± 3.69E-04	1.56E-03± 1.04E-04	1.24E-03± 6.04E-05	6.38E-03± 7.70E-04	1.07E-02± 3.56E-03	5.03E-03± 6.31E-04	6.19E-03± 7.24E-04	1.04E-02± 1.77E-03	2.81E-02± 2.56E-03	1.52E-03± 1.99E-04	8.51E-03± 3.43E-03
**Met**	8.37E-04± 2.34E-04	1.63E-04± 5.56E-06	1.39E-03± 3.43E-04	1.61E-04± 5.41E-06	1.0E-04± 0.00E-00	3.44E-04± 2.75E-05	4.04E-04± 1.07E-04	2.20E-03± 7.11E-04	9.08E-04± 6.91E-04	3.35E-04± 1.46E-05	2.14E-04± 7.50E-05	2.26E-03± 2.78E-02	3.33E-04± 1.65E-04	2.67E-04± 8.55E-05	7.63E-04± 1.23E-04
**Phe**	4.40E-03± 1.03E-03	1.14E-03± 1.82E-04	3.00E-03± 3.87E-05	4.13E-04± 8.83E-05	7.93E-04± 6.90E-06	6.93E-04± 6.86E-05	8.29E-04± 1.25E-04	3.93E-03± 4.65E-04	4.68E-03± 8.38E-04	2.60E-03± 2.98E-04	3.85E-04± 1.66E-05	3.31E-03± 2.92E-02	2.97E-03± 8.38E-04	5.94E-04± 8.90E-05	1.75E-03± 4.95E-04
**Pro**	1.52E-01± 1.34E-02	8.43E-02± 7.79E-03	1.43E-01± 1.59E-02	8.98E-03± 2.56E-03	1.02E-02± 1.10E-03	2.53E-02± 4.42E-03	3.55E-02± 1.80E-02	1.20E-01± 1.00E-02	1.00E-01± 4.06E-03	1.53E-01± 4.94E-03	2.44E-02± 5.05E-03	8.13E-02± 3.08E-02	2.07E-02± 1.26E-02	4.54E-02± 3.31E-02	6.47E-02± 2.28E-02
**Ser**	2.77E-03± 6.39E-05	5.84E-03± 5.66E-04	8.29E-03± 2.09E-03	2.15E-03± 2.04E-04	3.45E-03± 3.46E-04	4.47E-03± 7.93E-04	3.65E-03± 5.56E-04	1.16E-02± 4.44E-03	9.82E-03± 5.64E-03	3.43E-03± 3.84E-04	1.39E-03± 4.41E-04	1.18E-02± 7.03E-03	6.40E-03± 1.43E-03	6.41E-04± 1.57E-04	2.80E-03± 6.79E-04
**Trp**	3.65E-03± 4.93E-04	1.56E-03± 5.32E-05	1.58E-03± 1.60E-04	6.60E-04± 2.22E-05	6.54E-04± 1.44E-04	8.57E-04± 8.48E-05	7.09E-04± 2.04E-05	2.35E-03± 1.02E-04	2.71E-03± 1.33E-04	2.06E-03± 8.96E-05	1.40E-03± 3.69E-05	2.52E-03± 7.42E-03	2.21E-03± 7.59E-05	5.81E-04± 3.21E-04	2.00E-03± 3.30E-04
**Thr**	3.84E-03± 6.40E-04	3.16E-03± 2.03E-04	9.98E-03± 2.58E-03	1.07E-03± 1.79E-04	1.95E-03± 4.03E-04	2.44E-03± 4.33E-04	4.77E-03± 7.32E-04	1.13E-02± 1.58E-03	7.91E-03± 3.53E-03	3.44E-03± 8.32E-04	1.86E-03± 2.55E-04	1.66E-02± 7.70E-03	6.60E-03± 1.96E-03	4.29E-04± 6.99E-05	1.69E-03± 5.38E-04
**Tyr**	1.69E-02± 3.63E-03	2.96E-03± 9.77E-05	4.63E-03± 8.13E-04	7.81E-04± 2.63E-05	1.09E-03± 9.46E-06	1.24E-03± 1.10E-04	1.12E-03± 1.41E-04	4.89E-03± 2.86E-04	6.15E-03± 8.83E-04	7.88E-03± 6.40E-04	1.29E-03± 9.76E-05	9.76E-03± 5.78E-03	4.28E-03± 5.39E-04	1.94E-03± 6.47E-04	9.23E-03± 2.08E-03
**Val**	2.73E-03± 1.82E-05	2.21E-03± 2.35E-04	8.12E-03± 1.18E-03	5.69E-04± 1.92E-05	7.93E-04± 6.90E-06	1.46E-03± 2.65E-04	1.93E-03± 4.61E-04	6.43E-03± 9.90E-04	5.95E-03± 2.72E-03	1.88E-03± 1.71E-04	1.19E-03± 1.09E-04	7.32E-03± 5.76E-03	3.41E-03± 8.04E-04	1.27E-03± 5.92E-04	4.31E-03± 7.30E-04

Note: AA[b], free amino acids (g/100g flour). Amino acids content refers to mean ± standard deviation (n=3). Gly and Cys measurements were not included because of missing values or reading below detection level. B73 wt and B73 *o2* were included for comparison.

Table S5. Popping volume (ml) comparison and percentages of popped kernels between BC_2F_5 populations and popcorn parental lines

	t-test for Equality of Means								Percentages of popped kernels
	Mean Difference	Std. Deviation Difference	Std. Error Difference	95% Confidence Interval of the Difference: Lower	95% Confidence Interval of the Difference: Upper	t	df	Sig. (2-tailed)	
QPP1-1 vs P3	-18.33	5.77	3.33	-28.58	-8.09	-5.50	3.20	0.0099	96.14% vs 100%
**QPP1-2 vs P3**	**1.67**	**5.77**	**3.33**	**-8.58**	**11.91**	**0.50**	**3.20**	**0.6495**	
QPP1-3 vs P3	-12.67	6.67	3.85	-25.26	-0.07	-3.28	2.87	0.0493	
**QPP4-1 vs P3**	**-6.00**	**3.83**	**2.21**	**-12.18**	**0.18**	**-2.71**	**3.93**	**0.0544**	97.70% vs 100%
QPP4-2 vs P3	-35.00	4.07	2.35	-41.54	-28.46	-14.85	4.00	0.0001	
QPP4-3 vs P3	-14.00	4.97	2.87	-22.30	-5.70	-4.88	3.63	0.0104	
QPP2-1 vs P2	-24.83	11.10	6.41	-46.12	-3.55	-3.88	2.79	0.0345	94.12% vs 100%
QPP2-2 vs P2	-26.33	5.23	3.02	-35.76	-16.91	-8.72	3.11	0.0027	
**QPP2-3 vs P2**	**-10.00**	**7.00**	**4.04**	**-21.31**	**1.31**	**-2.47**	**3.92**	**0.0699**	
QPP6-1 vs P3	-69.00	6.79	3.92	-79.90	-58.10	-17.62	3.97	6.43E-05	100% vs 100%
QPP6-2 vs P3	-50.67	5.42	3.13	-60.03	-41.31	-16.20	3.37	0.0002	
QPP6-3 vs P3	-75.67	5.42	3.13	-85.03	-66.31	-24.20	3.37	6.72E-05	
QPP5-1 vs P5	-52.67	6.81	3.93	-63.60	-41.73	-13.401	3.9751	0.0002	90.68% vs 100%
QPP5-2 vs P5	-49.00	5.11	2.95	-60.81	-37.19	-16.644	2.1597	0.0026	
QPP5-3 vs P5	-53.33	5.23	3.02	-64.56	-42.11	-17.669	2.3701	0.0014	
QPP3-1 vs P9	-18.00	4.76	2.75	-26.25	-9.75	-6.5485	3.3483	0.0051	94.23% vs 100%
QPP3-2 vs P9	-27.33	3.22	1.86	-32.59	-22.08	-14.728	3.8059	0.0002	
QPP3-3 vs P9	-25.00	4.76	2.75	-33.25	-16.75	-9.0951	3.3483	0.0018	

Note. QPP1 (introgression from F_1 cross CML154Q x P3), QPP2 (introgression from F_1 cross CML154Q x P2), QPP3 (introgression from F_1 cross CML 154Q x P9), QPP4 (K0326Y x P3), QPP5 (K0326Y x P5), QPP6 (Tx807 x P3) and popcorn lines were used for small-scale popping analysis. Three different BC_2F_5 populations (indicated as QPP-1, -2 and -3) were used for each introgression to show variation for popping volume between BC_2F_5 ears. Three pools of kernels (3.33g each) were prepared from each ear as replicates. The total volume measured was recorded for comparison. Comparison was carried out using two sample t-test (Welch's t test). Highlighted in bold are the ones without significant difference in popping volume ($\alpha = 0.05$) which are QPP1-2, QPP4-1, QPP2-3. Welch's t-test adjusts the number of degree of freedom when the variances are thought not to be equal to each other. The average differences between QPP replicates and corresponding popcorn parental lines were shown in the Mean Difference column. The standard deviation of the average difference was shown in column Std. Deviation Difference. Std. Error of the average difference was shown in column Std. Error Difference. The lower and upper boundaries of the 95% confidence interval of the true mean difference was shown in column 95% of Confidence Interval of the Difference. The t statistic was obtained by dividing the mean difference by its standard error. Probability of obtaining a t statistic with its absolute value being equal to or greater than the obtained statistic was indicated in the Sig. (2-tailed) column. In the Percentages of popped kernels column, ratio of popped kernels were calculated as "Sum of popped kernels in three replicates" divided by "Sum of kernels in three replicates prepared for popping".
